# Nanotubes Growth by Self-Assembly of DNA Strands at Room Temperature

**DOI:** 10.1021/acsnano.4c17516

**Published:** 2025-05-08

**Authors:** Laura Bourdon, Syed Pavel Afrose, Siddharth Agarwal, Debajyoti Das, Rajat Singh, Aurélie Di Cicco, Daniel Lévy, Ayako Yamada, Damien Baigl, Elisa Franco

**Affiliations:** † PASTEUR, Department of Chemistry, 26909 École Normale Supérieure, PSL University, Sorbonne Université, CNRS, Paris 75005, France; ‡ Department of Mechanical and Aerospace Engineering, 8783 University of California at Los Angeles, 420 Westwood Plaza, Los Angeles 90095, California, United States; § Department of Medicine, David Geffen School of Medicine, University of California, Los Angeles 90095, Los Angeles, California, United States; ∥ Division of Digestive Diseases, David Geffen School of Medicine, 12222 University of California, Los Angeles 90095, Los Angeles, California, United States; ⊥ Comprehensive Liver Research Center at University of California, Los Angeles 90095, Los Angeles, California, United States; # Université PSL, Sorbonne Université, CNRS UMR168, Laboratoire Physico-Chimie Curie, 55216 Institut Curie, Paris 75005, France

**Keywords:** DNA nanotechnology, isothermal assembly, self-organization, DNA tile, synthetic cell, microdroplet, giant unilamellar vesicle

## Abstract

Artificial biomolecular nanotubes are a promising approach to building materials mimicking the capacity of the cellular cytoskeleton to grow and self-organize dynamically. Nucleic acid nanotechnology has demonstrated a variety of self-assembling nanotubes with programmable, robust features and morphological similarities to actual cytoskeleton components. However, their production typically requires thermal annealing, which not only poses a general constraint on their potential applications but is also incompatible with physiological conditions. Here, we demonstrate that DNA nanotubes can self-assemble from a simple mixture of five short DNA strands at constant room temperature, growing for extended periods of time in bulk conditions as well as under confinement. Assembly is achieved using a monovalent salt buffer, which ensures a faithful nanoscale arrangement and avoids nanotube aggregation. We observe the formation of individual nanotubes up to 20 days with a diameter of 22 ± 4 nm and length of several tens of micrometers. We finally encapsulate the strands in microsized compartments, such as water-in-oil microdroplets and giant unilamellar vesicles serving as simple cell models. Notably, nanotubes not only isothermally self-assemble directly inside the microcompartments but also self-organize into dynamic higher-order structures resembling rings and dynamic networks. Our study provides an advantageous method for *in situ* assembly of programmable biomolecular scaffolds and materials using synthetic DNA strands without requirements of thermal treatment.

## Graphical abstract

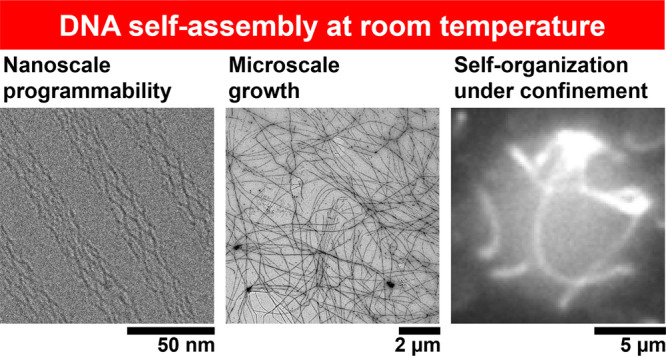

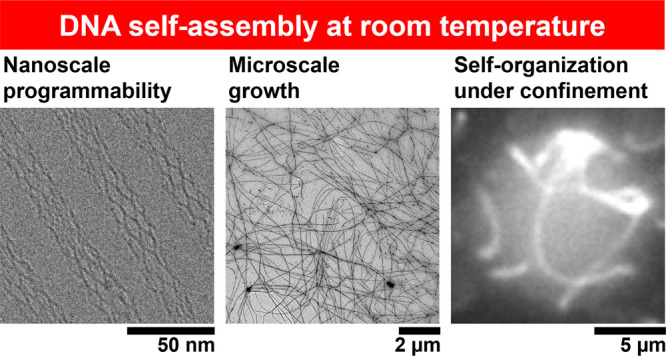

## Introduction

Through billions of years of evolution, nature came up with complex self-assembled architectures capable of performing functions crucial to the ability of biological machinery to operate seamlessly. These self-assembled structures are highly dynamic and possess the capability to grow, adapt, and reconfigure. For example, the cytoskeleton protein filaments are highly dynamic nonequilibrium self-assemblies that show remarkable spatiotemporal control over functions that regulate cell life-cycle, motility, and so forth.^1,2^ Creating synthetic self-assembled architectures that can demonstrate such dynamic and adaptive behavior can lead to materials with precise control over properties with potential application in diverse fields, from drug delivery to sensing.^3^ Nucleic acid (NA) nanotechnology is a powerful tool in this regard, as it offers great programmability and versatility through simple modification of strand sequences.^4^ By rationally designing the NA sequences according to Watson–Crick–Franklin base-pairing rules, parts of the different strands can be made complementary to each other, which can then hybridize to give rise to nearly arbitrary morphologies.^5,6^ Using the assembly of DNA tiles interacting through their sticky ends, self-assembled nanotubes have been produced,^7–9^ which resemble the cytoskeleton and actin filaments from a structural viewpoint.^9–14^

Current methods for manufacturing DNA nanostructures present limitations in terms of spatial features as well as adaptability in time. In the case of DNA origamis, for instance, the limitation in space is mainly due to the use of a scaffold that restricts the size of the final objects to around or below 100 nm.^5,15^ Several alternatives to this approach have been proposed, from scaffold-free self-assembly protocols^16^ to self-assembly of preformed origamis.^17–20^ The use of simple, repeated building blocks such as DNA tiles has made it possible to build lattices, filaments, and crystals reaching hundreds of micrometers in size,^7,21,22^ overcoming size limitations (at the expense of complexity). However, in the majority of cases, the formation of the targeted assembly has required the use of a thermal annealing step where the initial mixture containing the DNA strands is heated above the DNA melting temperature prior to a slow cooling down process. This creates a strong temporal constraint, as the resulting nanostructures are thermodynamically stable and usually do not continue to grow, adapt, or evolve in shape and size once annealing is completed.

Although reconfigurability of DNA nanostructures is possible by using strategies such as strand displacement,^23^ electrostatic suprafolding,^24^ and photocontrol,^25–29^ building structures capable of spontaneous growth and morphological adaptation is challenging yet highly desirable. Isothermal assembly is an interesting alternative to achieve dynamic structures because, by fixing the temperature, structures can be intrinsically more reconfigurable and free to evolve and potentially grow without any time constraints. Among the few reported methods for isothermal DNA self-assembly, for instance involving denaturing agents^30,31^ or high temperatures^32,33^ in magnesium-rich buffers, we opted for an approach based on a monovalent salt buffer where a variety of user-defined DNA nanostructures can not only be obtained at room temperature in mild conditions but also present a high degree of reconfigurability and adaptivity.^34^ This approach was previously applied to the isothermal self-assembly of DNA origamis and single-stranded tiles, leading to two- or three-dimensional objects no larger than 100 nm.^34^ Using strands coding for self-repeating units, nanogrids were produced, but defect-free assembly of a maximum of a few hundred nanometers was observed.

To investigate the potential for isothermal assembly and temporally sustained growth of larger DNA nanostructures, here we consider DNA nanotubes, a system consisting of five oligonucleotides forming a double-crossover (DX) tile that further assembles into cylindrical nanostructures.^7^ This design has been studied for many years as a model system to investigate nucleation, growth, and self-regulation of artificial polymers,^8,35,36^ and to build biomimetic endo- or exocytoskeletons,^10,11,14^ but nanotubes were always obtained either by thermal annealing or by the self-assembly of preannealed tiles, limiting possibilities of growth and evolution at fixed temperature. In this work, the oligonucleotides are mixed in a NaCl buffer, and we study their evolution over time at room temperature, without any thermal pre- or post-treatment (Figure 1A). Notably, we observe the autonomous formation of nanotubes growing into large dimensions and self-organizing into dynamic networks when confined in biomimetic compartments. Using fluorescence, transmission electron, and cryo-electron microscopy, we characterize the dynamic and structural features of these live-growing structures in bulk, in droplets, and in giant unilamellar vesicles (GUVs).

**Figure 1:**
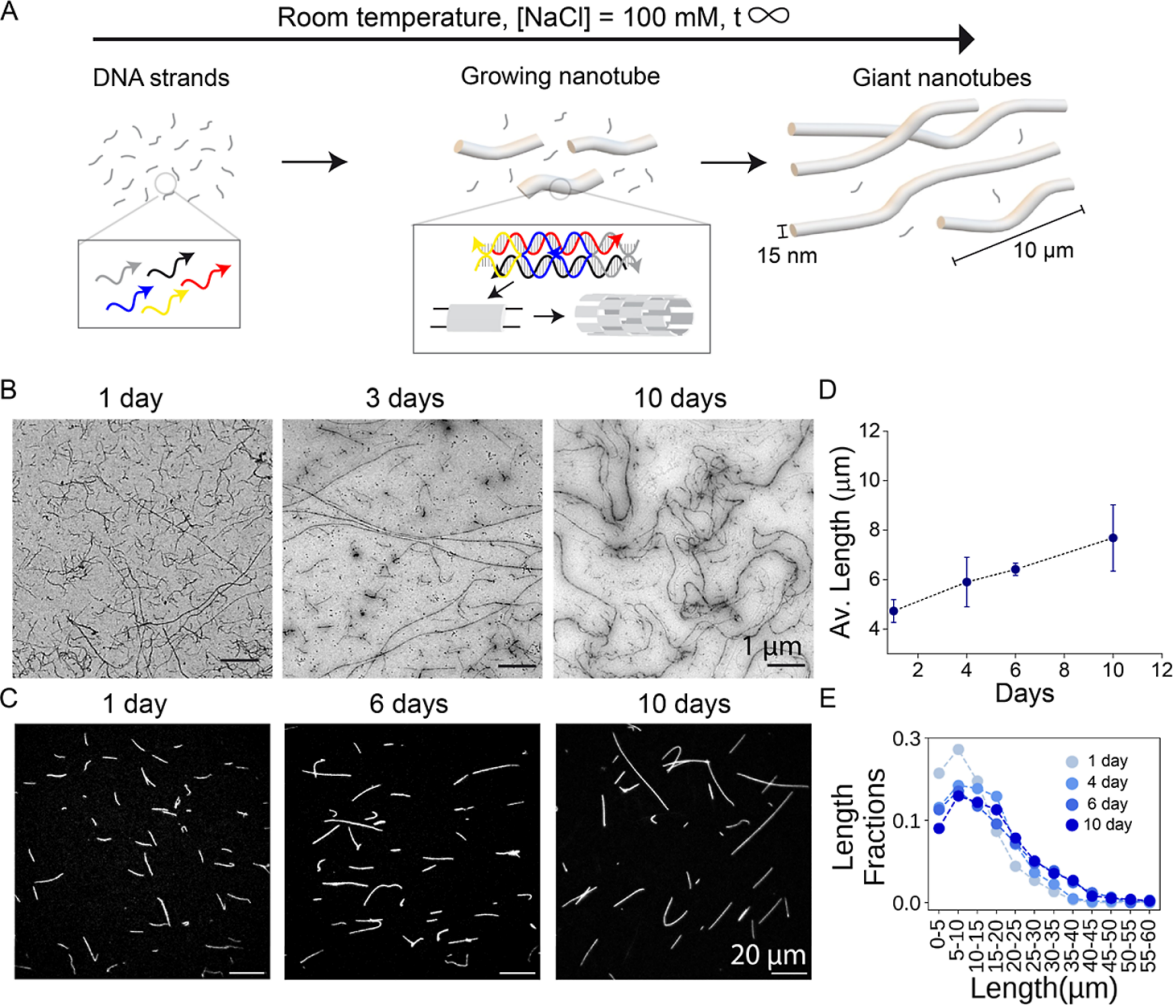
Formation of micrometer-long DNA nanotubes at constant room temperature. (A) A mixture of five DNA strands in TANa buffer (Trizma base 40 mM, acetic acid 20 mM, 100 mM NaCl) self-assemble isothermally into a self-repeating tile motif forming nanotubes, which grow in solution at room temperature over a prolonged period of time. (B) Representative transmission electron microscopy (TEM) images of nanotubes obtained after 1, 3, and 10 days of self-assembly at room temperature in TANa. Each strand concentration was 500 nM. (C) Representative fluorescence microscopy images of nanotubes obtained after 1, 6, and 10 days of self-assembly at room temperature in TANa. Each strand concentration was 1 μM. (D) Average length of the nanotubes calculated from fluorescence microscopy images and (E) length fraction histograms at different days. Error bars represent the standard deviation of the mean over 3 experimental replicates. Total nanotubes analyzed across triplicates at each time point: 1 day: 2903, 4 days: 3339, 6 days: 2224, and 10 days: 2402.

## Results

We chose DNA nanotubes assembling from DX tiles^7^ requiring the presence of only five short, distinct DNA strands designed to interact as depicted in Figure 1A, *left*. These strands form two DNA double helices held together as three of the strands (yellow, blue, and gray in Figure 1A) crossover from one helix to the other, forming two junctions that confer rigidity to the tile. The 5′-end of the strand in the center of the tile (blue in Figure 1A) was modified with the Cy3 dye to enable fluorescence microscopy observation. Tiles interact via complementary single stranded domains known as sticky ends (at the 5′ and 3′ end of the yellow and gray strands). The intertile crossover distance is chosen so that individual tiles bind to each other at an angle and thus form micrometer-scale tubular structures rather than flat lattices.^7,37^ Because these DNA nanotubes can be engineered to work as scaffolds with the capacity to respond to biochemical and physical stimuli, they are excellent components to build an “artificial cytoskeleton” for synthetic cells and responsive biomaterials.^9,10,14^ A notable limitation toward this has been the requirement to thermally anneal the DX tiles,^7,37^ which is at odds with the goal of building biocompatible and responsive systems operating at constant temperature. With the objective to build nanostructures that could emerge upon simple mixing of DNA strands, we placed the DNA strands in a buffer exclusively composed of monovalent cations, dubbed “TANa” buffer (Trizma base 40 mM, acetic acid 20 mM, and 100 mM NaCl), which was recently shown to enable successful assembly of complex DNA nanostructures at constant room temperature.^34^ Doing so, we questioned not only whether DNA nanotubes could assemble isothermally under these conditions but also how they would self-organize over time (Figure 1A, *middle*, *right*). To evaluate the performance and understand the advantage of using monovalent salts, we compared the results to those obtained with the magnesium-containing buffers conventionally used for DNA self-assembly, either TAMg (Trizma base, 40 mM; acetic acid, 20 mM; 12.5 mM MgCl_2_) or TAEMg (TAMg + 1 mM EDTA).

Direct incubation at room temperature of the five tile-forming DNA strands in TANa buffer resulted in the spontaneous formation of nanotubes, which were observed to freely grow in bulk without any thermal treatment before or during the assembly (Movie S1). TEM qualitatively revealed that short nanotubes slowly disappeared while longer ones became predominant (Figure 1B). To better quantify this process, nanotubes were adsorbed on glass, and their size distribution was established from automated image analysis. We found that growth started right after mixing the strands with a size evolution independent of the nanotube adsorption process (Figure S1). The system kept evolving for at least 10 days (Figure 1C), with an average nanotube length continuously increasing over time (Figure 1D). We also measured the fraction of each length range within the total length of nanotubes, indicating that as time progresses, the fraction of long nanotubes increases at the expense of shorter nanotubes (Figure 1E), confirming the TEM observations. We can thus conclude that, while the amount of DNA strands is fixed, the system continuously evolves over time, resulting in the overall isothermal growth of nanotubes that can individually reach lengths over 10 μm.

After 20 days of isothermal assembly in TANa, TEM images at different magnifications confirm the formation of distinct, well-separated nanotubes (Figure 2A, *top*). When the same isothermal formation experiment was done in TAMg buffer, replacing NaCl with 12.5 mM MgCl_2_, nanotubes could still be detected in the TEM images but were highly clustered in the form of micrometer-sized aggregates (Figure 2A, *bottom* and Movie S2) independent of the presence of EDTA (Figure S2). Since individual nanotubes can be obtained by thermal annealing in TAMg (Figure S3), the aggregates observed by isothermal assembly appear as kinetically trapped entities. To better understand the role of magnesium, we established the melting curves of nanotubes isothermally self-assembled in TANa to those thermally annealed with magnesium (Figure S4). We detected two characteristic melting temperatures (*T*_m_) in each case, attributed to the disassembly of the sticky ends (*T*_m_^1^) followed by that of the tiles (*T*_m_^2^). Although the tile melting temperature was unaffected by the buffer composition (*T*_m_^2^ = 62 °C in both cases), the sticky ends disassembled at a lower temperature in TANa (*T*_m_^1^ = 40 °C) when compared to that in the magnesium-rich buffer (*T*_m_^1^ = 43 °C). Typically adopted concentrations of magnesium thus stabilize the sticky-end bonds but likely also favor kinetic traps, resulting in aggregates when nanotubes are made by isothermal assembly at room temperature. In contrast, using TANa, we could assemble nanotubes without aggregation by incubating the mix of tile strands at constant temperatures ranging from 17 to 27 °C (Figure S5).

**Figure 2:**
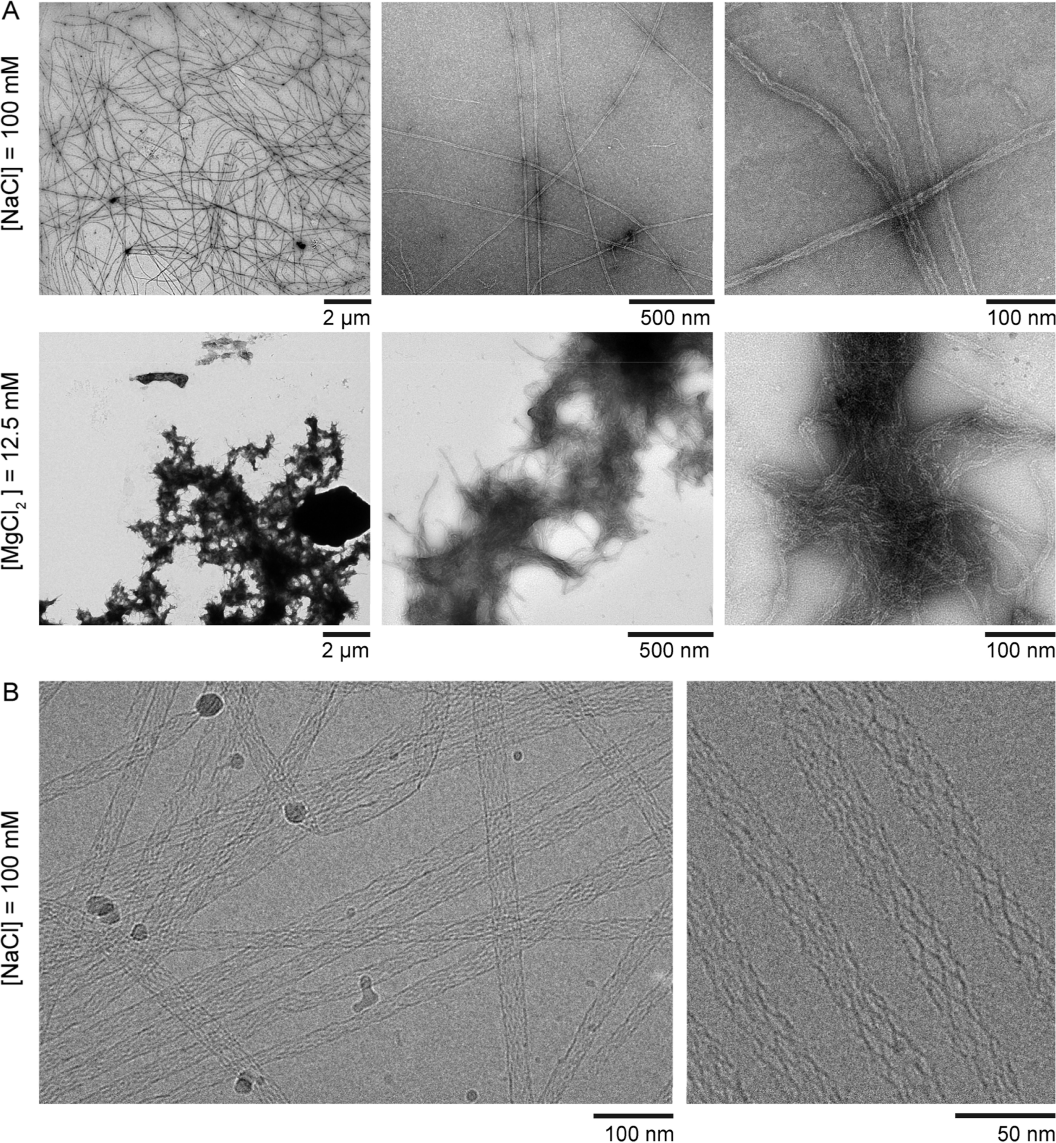
NaCl allows the isothermal assembly of individual, well-defined nanotubes at room temperature. (A) TEM images of DNA nanotube self-assembly at room temperature for 20 days in a TA buffered solution containing either 100 mM NaCl (TANa, top) or 12.5 mM MgCl_2_ (TAMg, bottom), at three magnifications. Each DNA strand was 500 nM. (B) Cryo-electron microscopy images of DNA nanotubes obtained by isothermal assembly at room temperature in TANa. Each DNA strand was 500 nM with assembly times of 10 days (left) and 5 h (bottom right).

Cryo-electron microscopy revealed a highly regular internal structure of the nanotubes with well-defined tiles aligned with the principal axis of the nanotubes (Figure 2B), confirming that the circumference of a nanotube generally includes 6–7 tiles, an internal structure comparable to that obtained by thermal annealing in the presence of magnesium (Figure S3).^7^ However, we noted that the diameter obtained by isothermal assembly in TANa (22 ± 4 nm, Figure S6A) was shifted to higher values when compared with the one obtained by thermal annealing in TAMg (12 ± 2 nm, Figure S6B). We also characterized the structure of nanotubes obtained by these two assembly methods. We found that the perimeter of hexagonal patterns created by assembled tiles remains constant (46 ± 3 and 46 ± 7 nm for TANa and TAMg, respectively). However, the width of these hexagonal patterns was significantly larger under isothermal assembly in TANa (7.6 ± 0.8 nm) when compared with thermal annealing in TAMg (5.3 ± 0.8 nm) (Figure S7). This suggests that the presence of magnesium in TAMg may be more effective than TANa in reducing electrostatic repulsion between parallel helices, which explains the smaller nanotube diameter. In summary, we have found that the high magnesium concentration in conventional thermal annealing buffers does not prevent the isothermal assembly of nanotubes *per se* but generates kinetically trapped clusters of highly aggregated nanotubes. In contrast, using a monovalent cation-rich buffer such as TANa enables isothermal growth of well-defined individual nanotubes with nanoscale organization and micrometric dimensions generally consistent with annealed samples.

To better characterize the capacity of TANa to enable isothermal emergence of nanotubes, we compared the growth dynamics in the first hours of assembly with that of nanotubes formed in conventional TAEMg buffer typically used for this purpose.^37^ Because isothermal assembly in the presence of Mg^2+^ induces nanotube aggregation, we set up a protocol in which strands 1, 2, 3, and 5 were preassembled, either by incubation (TANa) or thermal annealing (TAEMg), prior to introducing strand 4 (*t* = 0) and letting the system grow at room temperature (Figure 3A,D). For each time point, nanotubes were adsorbed on glass using the same protocol as in Figure 1C and their length distribution was established by image analysis. In TANa, the average nanotube length progressively increased with time, reaching up to 5.1 ± 0.8 μm at 24 h (Figures 3B and S8) accompanied by a progressive shift of the distribution (Figure 3C, *top*). Length fraction plots show that the contribution of short nanotubes diminishes over time as longer ones dominate (Figure 3C, *bottom*), consistent with what we observed on a longer time scale (Figure 1). In TAEMg, nanotubes also grew after adding strand 4 (Figures 3E and S8), but their average length plateaus after around 30 min, in striking contrast to the growth in TANa buffer. The early saturation was also evident from both the frequency-based and length fraction histograms (Figure 3F). To gain more insights into the growth mechanisms in TANa buffer when compared to TAEMg buffer, we plotted the cumulative complementary distribution function (CCDF) of the nanotube length in each case (Figure 3G–J). These CCDF plots estimate the likelihood to find a nanotube larger than a given value. DNA nanotube length is expected to follow an exponential distribution,^37^ whose CCDF is a straight line in a semilogarithmic plot, as confirmed in Figure 3G,I. When normalized with respect to its average, any exponential CCDF should collapse on a straight line with slope −1.^38^ While data for both TANa and TAEMg incubated nanotubes generally follow this trend (Figure 3H,J), TANa incubated nanotubes show more discrepancies with respect to the exponential model. In particular, during the initial phases of growth, the system is less likely to present long nanotubes when compared to the exponential case. A possible explanation for this behavior is that correctly formed TANa-assembled tiles may be initially less abundant, hindering polymerization; alternatively, nucleation events may be very frequent in TANa conditions due to the more dynamic interactions among DNA strands, which would result in a larger number of shorter nanotubes. The deviation from the exponential model is more pronounced when all five strands are incubated simultaneously in TANa buffer (Figure S1D,G). In this case, the system is more likely to produce long nanotubes at longer time scales. We hypothesize that the enhanced capacity of tiles to dynamically interact in TANa may promote the end-joining of existing nanotubes, resulting in overall longer assemblies.

**Figure 3:**
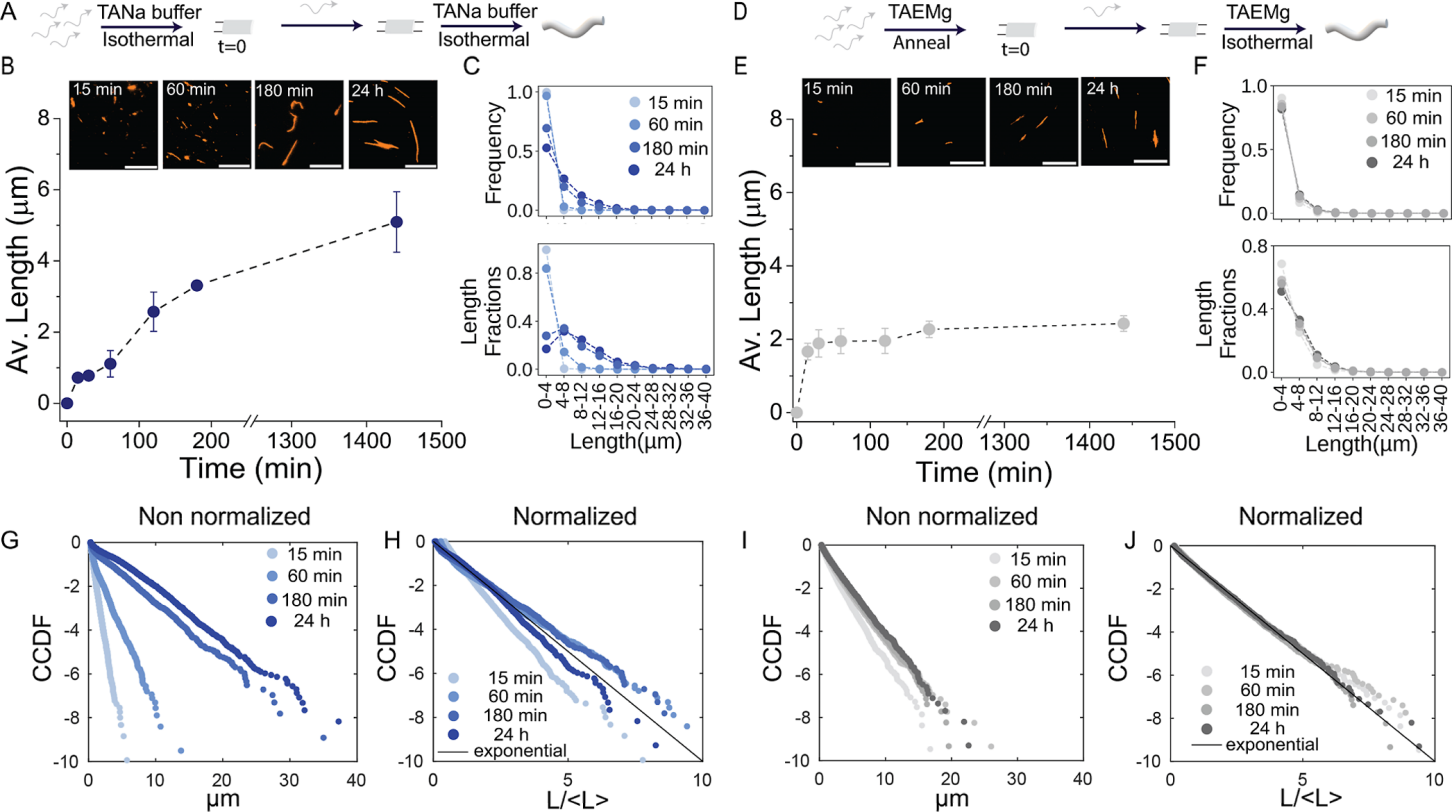
Growth of DNA nanotubes made from single-tile designs in TANa and TAEMg buffers. (A) Scheme showing the isothermal assembly process in TANa buffer, (B) microscopy images and average nanotube length at different time points, and (C) frequency and length fraction histograms. Total nanotubes analyzed across triplicates at each time point: 0 min: 0, 15 min: 10,349, 30 min: 6668, 60 min: 6698, 120 min: 5300, 180 min: 3708, and 24 h: 5301. (D) Scheme showing the isothermal assembly process of nanotubes in TAEMg buffer, (E) microscopy images and average nanotube length at different time points, and (F) frequency and length fraction histograms. Total nanotubes analyzed across triplicates at each time point: 0 min: 0, 15 min: 6466, 30 min: 4007, 60 min: 5732, 120 min: 7679, 180 min: 5644, and 24 h: 5467. (G–J) Normalized and non-normalized CCDF plots of nanotube length for samples in TANa (G,H) and in TAEMg (I,J). The concentration of each strand in these experiments is 1 μM. Scale bars = 10 μm. Error bars represent the standard deviation of the mean over 3 experimental replicates.

We then verified that a different nanotube variant, including two distinct tiles (a total of ten DNA strands), assembles correctly in TANa buffer (Figures S9 and S10). These tiles (dubbed SEp and REp by Rothemund^7^) have mutually complementary sticky ends that result in the assembly of nanotubes with parallel “rings” of distinct tiles (Figure S9A).^9,14^ Surprisingly, the average length of these nanotubes does not increase as much as in the single tile case, although trends for the length histograms, length fractions, and CCDF plots are consistent with the single tile case when compared with tiles annealed in TAEMg (Figures S11–S13). In general, it is known that the elongation of ′AB′-type polymers is sensitive to stoichiometric imbalance between the two monomer types.^39^ Further, the required tile pattern can compromise the likelihood of productive collision and binding of a given tile to a growing nanotube edge. The reduced probability of a growing nanotube encountering the appropriate subunits—whether tiles or fully formed rings—could further impede its growth. Specifically, since the nanotube now requires the correct pairing of tiles (i.e., tile 1 must bind with tile 2, and vice versa), the likelihood of encountering the ′right′ next segment is effectively halved compared to systems with a single tile design. Finally, we hypothesize a reduction of successful end-joining events due to “irregular” growth edges presenting incomplete parallel rings and potentially more frequent fragmentation due to joining defects. Interestingly, the TANa buffer allows the growth of the nanotubes with (Figures S9 and S10) or without (Figures S14–S16) prior incubation of the strands to form the tiles. Overall, we demonstrated that the TANa isothermal conditions allow a simple mix of five DNA strands to self-assemble into nanotubes that grow over days, exceeding the average length achievable with the conventional TAEMg assembly buffer.

DNA nanotubes are a promising scaffolding system for the development of composite biomaterials and synthetic cells.^9,10,14,40^ The possibility of assembling nanotubes in confinement at constant temperature, starting from the mere encapsulation of a few DNA strands, would drastically simplify protocols for building scaffolds in confinement as well as methods to control their emergence or dissolution in response to the simple release or sequestration of strands (through chemical reactions, material exchange, or other physical stimuli). We thus studied how TANa-assembled nanotubes self-organize when confined in microcompartments, starting with water-in-oil (W/O) emulsion droplets made of a fluorinated oil and biocompatible-surfactant mixture^9,41^ (Figure 4A). The five strands of the single tile design (100 nM in TANa) were encapsulated inside the droplets, and the system was incubated at room temperature. With time, nanotubes emerged and grew inside the droplets, forming structures that appeared as branched networks as a result of confinement (Figure 4B). In contrast, only linear structures were detected under bulk conditions (Movie S1 and Figures 1 and 2). After 2 days, dense networks were observed inside the droplets, suggesting continued nanotube growth as tiles polymerize. Growth in confinement also induced the formation of bent structures and ring-like morphologies (Figure 4C). Further, example time-lapse microscopy images and Supporting Information Videos (Figure S17 and Movie S3) show that nanotubes are highly mobile inside the droplets. When subjected to an increasing temperature, the droplet-encapsulated nanotubes progressively disappeared and could not be detected anymore at around 38 °C (Figure S18), in agreement with the melting temperature of sticky-end bonds measured in the bulk (Figure S4).

**Figure 4:**
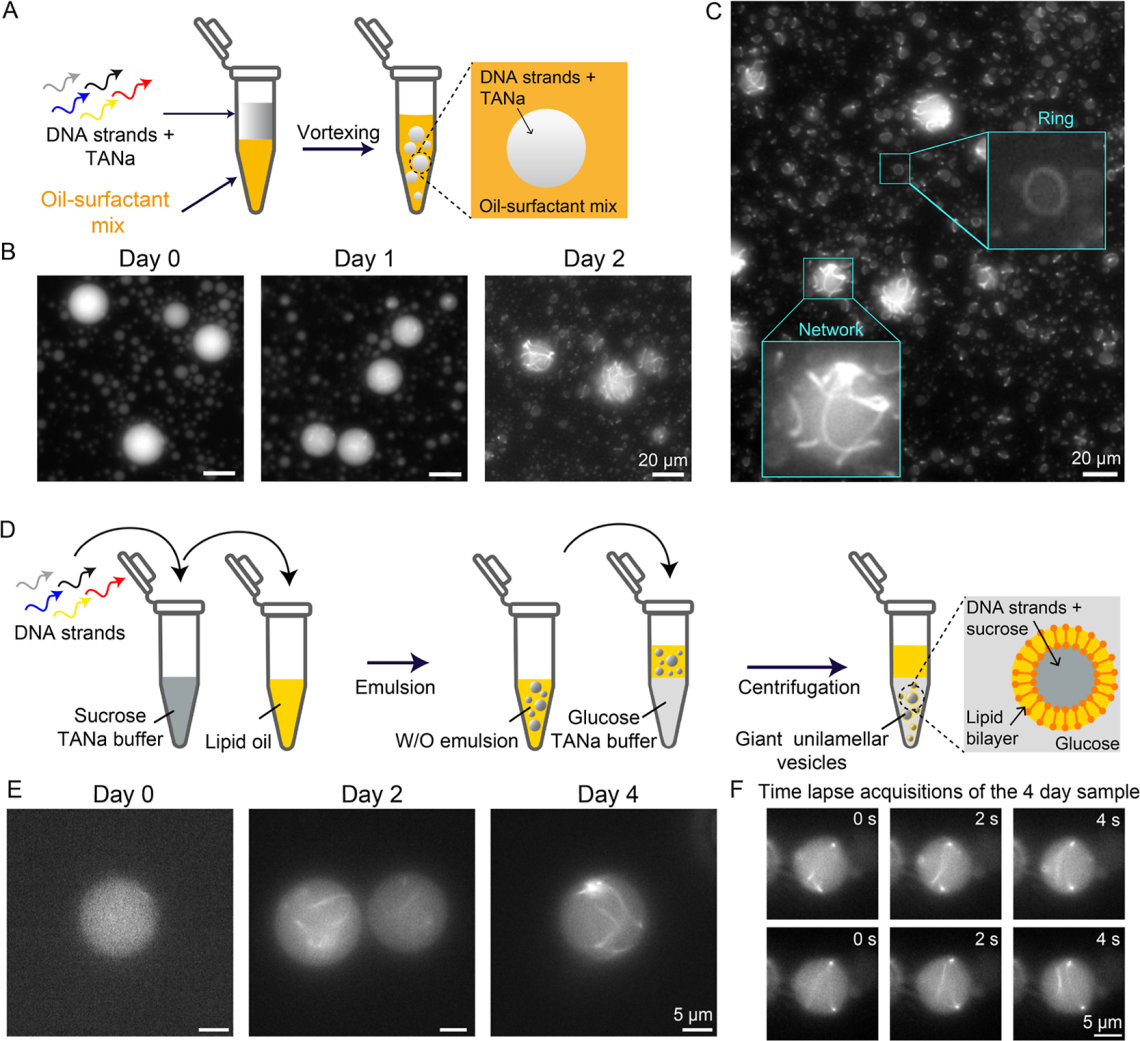
Isothermal growth and self-organization of DNA nanotubes in biomimetic confinement. (A) Scheme showing the protocol for encapsulation of DNA strands inside water-in-oil droplets. (B) Representative fluorescence microscopy images of nanotubes encapsulated in water-in-oil droplets in the presence of TANa buffer at different time points (each strand concentration is 100 nM). (C) Fluorescence microscopy image showing network and ring-like structure formation from the self-assembly of DNA strands inside droplets at room temperature. (D) Scheme showing the protocol for encapsulation of DNA strands inside giant unilamellar vesicles (GUVs) using TANa buffer (100 mM NaCl) and glucose as the outer medium. Each GUVs contains the DNA strands (each strand concentration is 500 nM) and sucrose (to ensure iso-osmotic conditions) in the same buffer and is kept at room temperature. (E) Representative fluorescence microscopy images of nanotubes growing and reorganizing inside the GUVs at different times after the DNA strands encapsulation. (F) Time lapse fluorescence microscopy images of a representative GUV taken after 4 days of encapsulation, showing the dynamics of the formed networks. Each row is a different time lapse acquisition starting at *t* = 0 taken with the sample incubated for 4 days after encapsulation.

Next, we asked if isothermal assembly can be extended to compartments that have a membrane, using giant unilamellar vesicles (GUVs) as closer mimics of synthetic cellular microenvironments. GUVs were prepared by adapting emulsion transfer protocols (Figure 4D and Methods for details).^42–44^ Contrary to previous works,^10,14^ we did not encapsulate preformed tiles or nanotubes, but directly the five strands (1–5, 500 nM each in TANa) in the GUVs, and let the system evolve over time. Encapsulation of the DNA strands within the lipid membrane was successful, as indicated by the observation of fluorescence (Cy3-labeled strand 3) in the vesicle interior (Figure 4E) and its membrane (Figure S19). We subsequently monitored the isothermal growth of nanotubes inside the GUVs. After 2 days, individual nanotubes could be observed inside the vesicles, showing that isothermal growth was also occurring in these cell-sized microenvironments. From day 4, we observed the emergence of networks and ring-like morphologies (Figure 4E,F and Movie S4), recapitulating the behavior observed in microdroplets. Spinning disk confocal microscopy images of the same vesicle taken at days 1 and 5 revealed the intensification and an overall growth of the encapsulated nanotube network over time (Figure S20 and Movie S5). Interestingly, similar reorganization of DNA nanotubes into cytoskeleton-like assemblies inside GUVs has been recently reported, but it always involves the addition of condensing agents, such as crowding agents or high concentrations of Mg^2+^.^10,14^ Here, we find that the combination of isothermal self-assembly from molecular bricks (DNA strands) with confinement is enough to generate such dynamic self-organization. Overall, the isothermal growth of DNA nanotubes inside W/O droplets and vesicles suggests that they are a viable approach to building dynamic, responsive scaffolds in synthetic cells and living materials. Isothermally assembling DNA nanotubes can form adaptive architectures that may be engineered to achieve spatiotemporal control over compartment functions and mimic the dynamic and highly reconfigurable nature of the cytoskeleton components.

## Conclusions

We demonstrated the isothermal growth of DNA nanotubes at room temperature using a Mg-free monovalent NaCl containing buffer (TANa) without the need for an annealing step. Starting from a mixture of 5 different DNA strands, nanotubes with the desired structure grow for days, as verified through different microscopic techniques such as TEM, cryo-electron microscopy, and fluorescence microscopy. Frequency and length fraction analysis shows the gradual disappearance of smaller nanotubes and the appearance of longer nanotubes over time, likely because joining events are more favorable when compared to the case of assembly using divalent cations that favor kinetic trapping. This results in sustained growth over days, allowing a simple molecular program of a few elementary self-assembling bricks to reach mesoscopic dimensions, while ensuring near-flawless assembly at the nanoscale level. This characteristic constitutes a promising asset for the design of future self-assembled smart materials capable of adaptation and self-healing.

The NaCl-based buffer allowed the nanotubes to self-assemble isothermally inside compartments (W/O droplets as well as GUVs serving as cell models), where they not only grow but also spontaneously reorganize, without the addition of any cross-linking or condensing agents, into dynamic higher-order assemblies such as networks and ring-like structures, forming a valuable example of confinement-induced self-organization in a synthetic cell-mimicking system. In our study, nanotubes are physically isolated from the environment once encapsulated in vesicles and, thus, must eventually reach an equilibrium length distribution. However, the inclusion of protein or DNA origami nanopores could establish the exchange of NAs or fuel molecules to further sustain growth and build out-of-equilibrium systems.^45,46^

We found that the use of TANa buffer caused a decrease in the nanotube melting temperature relative to that when they were grown in TAEMg. This suggests a lower thermodynamic stability of the nanotubes formed in TANa buffer: this is an advantage in terms of dynamic reconfigurability of the structures but could pose a challenge for growing nanotubes at physiological temperatures in biological applications.^47^ However, the stability could be easily improved by increasing the length of the tile sticky ends. Another approach could be to optimize the buffer composition to include limited amounts of MgCl_2_ to enhance thermal stability. Inclusion of MgCl_2_ is often also required for proper enzyme activity, for example, in the case of in vitro RNA transcription that has been used to generate adaptive responses in nanotube systems.^8^ Buffers with a mixture of monovalent and divalent cations may enable isothermal assembly as well as stability in the presence of enzymatic reactions.

The conditions used here to assemble DNA nanotubes also enable the isothermal assembly of more complex DNA structures, including various types of DNA origami.^34^ For this reason, our work indicates that it may be possible to assemble at constant temperature DNA systems that simultaneously take advantage of both origami methods,^48^ known for achieving arbitrary nanometer scale patterning, as well as tiling methods,^49^ which easily produce micrometer scale assemblies. Strand- and tile-based assemblies also constitute convenient ingredients for algorithmic self-assembly,^50^ allowing to program higher-order morphologies through computation. The combination of all of these traits may lead to the development of multiscale DNA materials rivaling the complexity and adaptability of biological assemblies. These materials may in turn be made responsive to strand displacement networks^51^ or to physical inputs^27^ to achieve an even greater level of adaptation.

## Methods

### TANa, TAMg, and TAEMg Buffers

The DNA oligonucleotides were mixed in a buffered solution containing 40 mM Trizma-base, 20 mM acetic acid, and either 12.5 mM MgCl_2_ for the TAMg buffer, or 100 mM NaCl for the TANa buffer. For the assembly studies with thermally annealed tiles, we used TAEMg buffer containing 40 mM Trizma-base, 20 mM acetic acid, 12.5 mM MgCl_2_, and 1 mM EDTA.

### Nanotube Self-Assembly

DNA nanotube sequences for each strand correspond to the single tile (SEs) and two-tile (REp + SEp) designs by Rothemund et al.^7^ and are listed in Supporting Information, Section 1. Each tile includes five DNA strands, which were mixed to a final concentration of either 500 nM or 1 μM in the desired buffer (TANa, TAMg, or TAEMg).

For isothermal self-assembly, strands were mixed at the appropriate concentration in the TANa buffer, and the solution was kept at room temperature (between 20 and 25 °C) and protected from light. For two tile experiments, strands for tile REp and SEp were either all mixed at once or separately incubated (REp or SEp) at room temperature in DNA Lo-bind tubes and then mixed (target 1 μM tile concentration for each tile) to initiate nanotube formation.

For thermally annealed self-assembly, strands were mixed at the appropriate concentration in TAMg or TAEMg, and the samples were annealed using a thermal cycler by heating to 90 °C and cooling to 25 °C over a 6 h period. For experiments involving the two-tile system, tile REp and tile SEp were annealed separately and then mixed (target 1 μM concentration for each tile) to initiate nanotube formation.

### Transmission Electron Microscopy

The DNA nanotubes were deposited on a plasma-treated 200 mesh copper grid that supports a carbon film (Ted Pella, USA). For the fixation, 10 μL of solution containing the nanotubes was left for 3 min on the grid. The solution was removed by blotting with filter paper from one side of the grid. Fixed DNA nanotubes were stained with 2% uranyl acetate (UA) solution in two steps: 5 μL of UA solution was first deposited on the grid and directly blotted, then 15 μL of UA solution was deposited and left for 60 s before being blotted. The grids were observed by using a JEOL microscope equipped with a GATAN camera at 200 kV.

### Cryo-Electron Microscopy

4 μL of undiluted solution of nanotubes in TANa or 20-fold diluted solution of nanotubes in TAMg were deposited on glow-discharged carbon-Formvar lacey grids (Ted Pella, USA), blotted from the back side, and flash frozen in liquid ethane with an EM-GP2 Leica plunger at 80% humidity. Cryo-EM images were acquired with a Glacios cryo-electron microscope (Thermo Fisher, USA) operating at 200 kV with a falcon IV camera and in low dose mode.

### DNA Nanotube Melting Characterization by UV–Vis Spectroscopy

Melting curves were established by using a Cary 300 (Agilent Technologies) UV–visible spectrophotometer equipped with a Peltier temperature controller. Measurements were performed with 100 μL of sample placed in a quartz cell (Submicrocell quartz 10 mm, 50 μL, Agilent Technologies). We measured 6 replicate absorptions at 260 nm every 1 °C, from 20 °C until 90 °C. Melting temperatures were detected by plotting the derivative of absorbance with temperature.

### Fluorescence Microscopy

DNA nanotubes that are labeled with Cy3 dye were observed using either a ZEISS Observer Z1 microscope equipped with a Plan-Apochromat 100x/1.4 NA oil objective and a Semrock LED-YFP-A (emission 509/22 nm and excitation 544/24 nm) or an inverted microscope (Nikon Eclipse TI-E) with a Nikon Plan Fluor 60X/1.4 NA oil immersion objective.

For imaging DNA nanotubes in bulk, samples were placed in a 6 mm chamber composed of a PDMS well, which was closed with two glass coverslips (Movies S1 and S2). To image DNA nanotubes on surfaces, the nanotube solution was diluted and deposited on a Fisherbrand microscope glass slide (cat. no. 125442, 1 mm; size: 75 × 25 mm) and gently covered with Fisherbrand Cover Glasses (cat. no. 12541B). Nanotubes incubated in TANa generally adsorb on the glass slide; however, we noted variability of adsorption depending on the glass slide batch. Poor adsorption is associated with nanotubes that can partially move during imaging, which hinders consistent length measurements. To increase adsorption on the glass surface, it is beneficial to add a small amount of MgCl_2_ (5 mM) to the diluted sample prior to imaging. To probe whether the addition of MgCl_2_ affects the growth of the nanotubes, a control experiment was done with the 1 tile design nanotubes by adding MgCl_2_ before imaging at each time point. This yielded similar nanotube lengths for samples with and without the addition of MgCl_2_ (Figure S1), proving that the addition of MgCl_2_ right before imaging did not influence the nanotube lengths. The analysis and the calculation of the average nanotube length were done using code developed in-house from fluorescence images taken with an exposure time of 90 ms.

Droplet-encapsulated nanotubes were imaged in an Ibidi chamber (μ-Slide VI 0.4, uncoated) with the inputs to the channels sealed with vacuum grease (Dow Corning) and Fisherbrand Cover Glasses (cat. no. 12541B) to prevent evaporation. For imaging the GUVs, we used an 8 mm PDMS chamber enclosed by two glass coverslips. The chambers were previously passivated with 0.5% casein PBS solution and washed with the buffer containing the vesicles (called the “outside solution”) to minimize surface interactions. The fluorescence from the Cy3-labeled DNA and from the RhodamineB-labeled lipid membrane was measured using a microscope (ZEISS Observer Z1) equipped with a Plan-Apochromat 100x/1.4 NA oil objective.

### Measurement of Average Nanotube Lengths

Images have their brightness and contrast adjusted for clarity for the time course series images presented in the text and Supporting Information.

We extracted DNA nanotube length measurements from epifluorescence micrographs using a custom Python script available on Github:https://github.com/klockemel/DNA-Nanotube-Lengths

This script implements several Python packages, including scikit-image, pandas, and others.^52,53^ Generally, a threshold is applied to micrographs of fluorescently labeled DNA nanotubes affixed to a glass slide, and the lengths of nonintersecting nanotubes are measured. Each image is read into the script from either a 16 bit tif or an nd2 image, where nd2 is the proprietary image format generated by the Nikon NIS-Elements Software. To minimize the influence of image background issues in measurements, such as uneven illumination, an approximation of the image background is generated and subtracted from the original image. The image background is generated by applying a median filter to the image to remove small bright features. The footprint parameter for the median filter was a disk of radius 10–20 pixels, where larger radii result in a more smoothed image and longer script run times. This background image is subtracted from the raw image, which has been smoothed with a Gaussian filter. The Gaussian filter prevents random noise within the image from distorting the detection of the nanotubes. The result is an image in which the nanotubes can be more clearly distinguishable from the background.

Following background subtraction, a threshold is applied to separate the nanotubes from the image background. We used the Otsu, Yen, or Triangle thresholds from the scikit-image library. After thresholding the image, the binarized result is thinned such that each feature is 1 pixel thick using the skimage.morphology.thin function. Any features less than 3 pixels long are removed, as it is unclear if those objects are nanotubes. Finally, intersecting features are removed using a branch-point detection script. Lengths of nanotubes are then measured using the skimage.measure.regionprops function. All user–input parameters for each image are saved in a .csv file, and a diagnostic image with measured nanotubes is generated.

### Measurement of Nanotube Diameters and Tile Sizes

Nanotube diameters and tile sizes (width and perimeter) were measured on Cryo-EM images using ImageJ. For consistency of the tile width and perimeter measurements, only fully visible tiles positioned at the center of the nanotubes were considered.

### Encapsulation of Oligonucleotides Inside Water-In-Oil Droplets

Nanotube encapsulation in W/O droplets was achieved by combining 80 μL of oil-surfactant mix and 20 μL of aqueous phase containing the oligonucleotides of the desired concentration. Emulsion droplets were formed by vortexing for 50 s on a benchtop vortexer. The milky appearance of the sample indicated successful emulsification. Timing and speed of vortexing affect the average droplet size. For imaging, the sample was allowed to settle for 5–7 min, and aliquots were drawn from below the dense layer at the top of the sample to avoid an excessive concentration of overlapping W/O droplets in the field of view. The sample was placed in Ibidi chamber slides (μ-Slide VI 0.4). To prevent contamination and evaporation of the sample, the chamber wells were covered with a glass coverslip sealed using vacuum grease (Dow Corning). Since the assembly process begins as soon as the encapsulation step is completed, imaging via fluorescence microscopy was started immediately after loading the sample into the imaging chamber.

To estimate the melting temperature of the nanotubes inside the droplets, a fixed field of view was chosen, and the temperature was increased 1 °C at a time. After the desired temperature was reached, the sample was equilibrated at that temperature for 10 min, and images were taken.

### Encapsulation of Oligonucleotides Inside GUVs

The protocol to form lipid vesicles by phase transfer was adapted from previous reports.^42–44^ A DOPC lipid film with or without 0.5% Liss Rhod PE (for fluorescently labeled vesicles) was resuspended in mineral oil by sonication for 1 h, to a final concentration of 0.7 mg.mL^–1^. The solution outside the vesicles (outside solution) was made of 40 mM Trizma base, 20 mM acetic acid, 100 mM NaCl, and 230 mM glucose. The solution inside the vesicles (inner solution) had the same composition, except that the glucose was replaced by 230 mM sucrose and that the solution contained 500 nM of the five DNA strands from the single tile nanotube design. The osmolarity of both solutions was measured to be around 500 mOsm. The sucrose solution, which had a higher osmolarity, was slightly diluted with water to reach a similar osmolarity (ΔOsm <5 mOsm). The sucrose solution containing the DNA (inner solution) was emulsified by pipetting in the lipid oil solution, to a ratio of 1:30. The emulsion was slowly added in a lipid oil solution that was placed on top of the glucose solution (outside solution). The W/O droplets were left sedimented for 5 min at the W/O interface, before being centrifuged at 1000 rcf for 3 min. The oil solution was removed, and the outside solution containing the vesicles was observed by fluorescence microscopy.

### Spinning Disk Confocal Microscopy

Images were acquired using optically demodulated structured illumination super-resolution microscopy (SIM-Live SR). GUVs were imaged on a Nikon Ti2 CSU-W1 spinning disc confocal microscope equipped with a FI60 Plan Apochromat Lambda D 100× Oil Immersion Objective (N.A. 1.45, W.D. 0.13 mm, F.O.V. Twenty-five mm, DIC, Spring Loaded). Image acquisition was performed using a Kinetix 22 back-illuminated sCMOS camera (C-mount, 22 mm FOV, 2400 × 2400 resolution, 83 FPS @ 16 bit, 6.5 μm pixel size). The effective image pixel sizes were 0.107 and 0.065 μm, respectively.

Red fluorescence was excited using a 560 nm laser, with emission collected through a quad-pass dichroic beam splitter (Di01-T405/488/568/647). The spinning disc was configured with the Live SR super-resolution modality and further enhanced using 3D deconvolution and Denoise.ai to improve both axial and lateral resolution, achieving subcellular detail down to ∼105 nm.

To maintain high-resolution imaging across varying depths, extended depth of focus algorithms were applied to each Z-stack, generating composite images in which each pixel was in focus. This approach preserved transverse resolution across an extended depth range that exceeded the theoretical DOF. Movies were generated using the NIS elements movie maker plugin and rendered as Depth coded maximum intensity projection to capture the nanotubes within the GUVs.

All images were acquired under identical imaging conditions and exposure settings across the experiments. Postacquisition processing was performed uniformly by using consistent parameters and metrics.

### Quantification of Nanotubes Length

Individual image files were processed and visualized using a 3D volume viewer, followed by segmentation using Segment.ai. Manual pruning was performed postsegmentation to ensure accuracy. A 3D binary mask was generated based on the segmented structures to enable precise quantification of nanotubes. The 3D length of individual nanotubes was measured by using the Length 3D function within the General Analysis 3 module. Objects shorter than 0.3 μm were excluded from the final analysis under both experimental conditions.

Accurate measurement of structures moving along the *Z*-axis is inherently challenging due to potential motion artifacts, variability in speed, and limitations in resolution. Methods such as phase-shift profilometry are susceptible to these artifacts, requiring careful consideration during the analysis. As a result, 3D length measurements were utilized to assess the relative distribution and frequency of nanotubes between day 1 and day 5, rather than claiming absolute lengths, ensuring consistency and minimizing measurement inaccuracies due to motion artifacts.

## Supplementary Material













## Notes

Nanotube length analysis for the isothermal assembly in TANa with all 5 strands added together; isothermal assembly in TAEMg; cryo-EM images of nanotubes obtained by thermal annealing in TAMg; melting curves of DNA nanotubes; effect of temperature; histograms of DNA nanotubes diameters; tile width distributions; nanotubes obtained by isothermal addition of the fifth strand to preassembled tiles; isothermal assembly of two-tile nanotubes in TANa with preassembled tiles; formation of two-tile nanotubes in TAEMg with preannealed tiles; complementary distributions for two-tile nanotubes; isothermal assembly of two-tile nanotubes in TANa when all ten strands were added together; timelapse images of single tile nanotubes obtained by isothermal assembly in microdroplets; nanotubes in microdroplets exposed to different temperatures; fluorescence microscopy image of DNA strands forming nanotubes in a GUV with a dyed membrane; and spinning disk confocal analysis of DNA nanotubes growing in GUVs (PDF)
Real-time fluorescence video of the self-assembly of DNA nanotubes in TANa buffer at room temperature (MP4)
Real-time fluorescence video of DNA assemblies obtained from the one-tile design in TAMg buffer at room temperature for 10 days (MP4)
Real-time fluorescence video of the self-assembly and self-organization of DNA nanotubes inside W/O droplets at room temperature (AVI)
Real-time fluorescence video of the self-assembly and self-organization of DNA nanotubes inside GUVs at room temperature (MP4)
3D reconstruction of nanotubes encapsulated inside a GUV on day 1 and day 5 across the *z*- axis (MP4)

## References

[1] Hess H., Ross J. L. (2017). Non-Equilibrium Assembly of Microtubules: From Molecules
to Autonomous Chemical Robots. Chem. Soc. Rev..

[2] Gasic I., Mitchison T. J. (2019). Autoregulation
and Repair in Microtubule Homeostasis. Curr.
Opin. Cell Biol..

[3] Whitesides G. M., Grzybowski B. (2002). Self-Assembly
at All Scales. Science.

[4] Seeman N. C., Sleiman H. F. (2017). DNA Nanotechnology. Nat. Rev.
Mater..

[5] Rothemund P. W. K. (2006). Folding
DNA to Create Nanoscale Shapes and Patterns. Nature.

[6] Hong F., Zhang F., Liu Y., Yan H. (2017). DNA Origami: Scaffolds
for Creating Higher Order Structures. Chem.
Rev..

[7] Rothemund P. W. K., Ekani-Nkodo A., Papadakis N., Kumar A., Fygenson D. K., Winfree E. (2004). Design and Characterization of Programmable DNA Nanotubes. J. Am. Chem. Soc..

[8] Green L. N., Subramanian H. K. K., Mardanlou V., Kim J., Hariadi R. F., Franco E. (2019). Autonomous Dynamic Control of DNA Nanostructure Self-Assembly. Nat. Chem..

[9] Agarwal S., Klocke M. A., Pungchai P. E., Franco E. (2021). Dynamic Self-Assembly
of Compartmentalized DNA Nanotubes. Nat. Commun..

[10] Arulkumaran N., Singer M., Howorka S., Burns J. R. (2023). Creating Complex
Protocells and Prototissues Using Simple DNA Building Blocks. Nat. Commun..

[11] Illig M., Jahnke K., Weise L. P., Scheffold M., Mersdorf U., Drechsler H., Zhang Y., Diez S., Kierfeld J., Göpfrich K. (2024). Triggered
Contraction of Self-Assembled
Micron-Scale DNA Nanotube Rings. Nat. Commun..

[12] Glaser M., Schnauß J., Tschirner T., Schmidt B. U. S., Moebius-Winkler M., Käs J. A., Smith D. M. (2016). Self-Assembly of Hierarchically Ordered
Structures in DNA Nanotube Systems. New J. Phys..

[13] Stenke L. J., Saccà B. (2023). Design, Mechanical
Properties, and Dynamics of Synthetic
DNA Filaments. Bioconjugate Chem..

[14] Jahnke K., Huth V., Mersdorf U., Liu N., Göpfrich K. (2022). Bottom-Up
Assembly of Synthetic Cells with a DNA Cytoskeleton. ACS Nano.

[15] Douglas S. M., Dietz H., Liedl T., Högberg B., Graf F., Shih W. M. (2009). Self-Assembly of DNA into Nanoscale
Three-Dimensional Shapes. Nature.

[16] Yan H., Park S. H., Finkelstein G., Reif J. H., LaBean T. H. (2003). DNA-Templated
Self-Assembly of Protein Arrays and Highly Conductive Nanowires. Science.

[17] Woo S., Rothemund P. W. K. (2014). Self-Assembly of Two-Dimensional DNA Origami Lattices
Using Cation-Controlled Surface Diffusion. Nat.
Commun..

[18] Suzuki Y., Endo M., Sugiyama H. (2015). Lipid-Bilayer-Assisted Two-Dimensional
Self-Assembly of DNA Origami Nanostructures. Nat. Commun..

[19] Tikhomirov G., Petersen P., Qian L. (2017). Fractal Assembly of Micrometre-Scale
DNA Origami Arrays with Arbitrary Patterns. Nature.

[20] Wagenbauer K. F., Sigl C., Dietz H. (2017). Gigadalton-Scale
Shape-Programmable
DNA Assemblies. Nature.

[21] Paukstelis P. J., Nowakowski J., Birktoft J. J., Seeman N. C. (2004). Crystal
Structure
of a Continuous Three-Dimensional DNA Lattice. Chem. Biol..

[22] Lo P. K., Karam P., Aldaye F. A., McLaughlin C. K., Hamblin G. D., Cosa G., Sleiman H. F. (2010). Loading and Selective
Release of Cargo in DNA Nanotubes with Longitudinal Variation. Nat. Chem..

[23] Yurke B., Turberfield A. J., Mills A. P., Simmel F. C., Neumann J. L. (2000). A DNA-Fuelled Molecular Machine Made of DNA. Nature.

[24] Nakazawa K., El Fakih F., Jallet V., Rossi-Gendron C., Mariconti M., Chocron L., Hishida M., Saito K., Morel M., Rudiuk S., Baigl D. (2021). Reversible Supra-Folding
of User-Programmed Functional DNA Nanostructures on Fuzzy Cationic
Substrates. Angew. Chem., Int. Ed. Engl..

[25] Yang Y., Endo M., Hidaka K., Sugiyama H. (2012). Photo-Controllable
DNA Origami Nanostructures Assembling into Predesigned Multiorientational
Patterns. J. Am. Chem. Soc..

[26] Willner E. M., Kamada Y., Suzuki Y., Emura T., Hidaka K., Dietz H., Sugiyama H., Endo M. (2017). Single-Molecule Observation
of the Photoregulated Conformational Dynamics of DNA Origami Nanoscissors. Angew. Chem., Int. Ed. Engl..

[27] Bergen A., Rudiuk S., Morel M., Le Saux T., Ihmels H., Baigl D. (2016). Photodependent Melting of Unmodified
DNA Using a Photosensitive Intercalator:
A New and Generic Tool for Photoreversible Assembly of DNA Nanostructures
at Constant Temperature. Nano Lett..

[28] Zhou L., Retailleau P., Morel M., Rudiuk S., Baigl D. (2019). Photoswitchable
Fluorescent Crystals Obtained by the Photoreversible Coassembly of
a Nucleobase and an Azobenzene Intercalator. J. Am. Chem. Soc..

[29] Agarwal S., Dizani M., Osmanovic D., Franco E. (2023). Light-Controlled Growth
of DNA Organelles in Synthetic Cells. Interface
Focus.

[30] Jungmann R., Liedl T., Sobey T. L., Shih W., Simmel F. C. (2008). Isothermal
Assembly of DNA Origami Structures Using Denaturing Agents. J. Am. Chem. Soc..

[31] Zhang Z., Song J., Besenbacher F., Dong M., Gothelf K. V. (2013). Self-Assembly
of DNA Origami and Single-Stranded Tile Structures at Room Temperature. Angew. Chem., Int. Ed. Engl..

[32] Sobczak J.-P. J., Martin T. G., Gerling T., Dietz H. (2012). Rapid Folding of DNA
into Nanoscale Shapes at Constant Temperature. Science.

[33] Song J., Li Z., Wang P., Meyer T., Mao C., Ke Y. (2017). Reconfiguration
of DNA Molecular Arrays Driven by Information Relay. Science.

[34] Rossi-Gendron C., El Fakih F., Bourdon L., Nakazawa K., Finkel J., Triomphe N., Chocron L., Endo M., Sugiyama H., Bellot G., Morel M., Rudiuk S., Baigl D. (2023). Isothermal
Self-Assembly of Multicomponent and Evolutive DNA Nanostructures. Nat. Nanotechnol..

[35] Barish R. D., Schulman R., Rothemund P. W. K., Winfree E. (2009). An Information-Bearing
Seed for Nucleating Algorithmic Self-Assembly. Proc. Natl. Acad. Sci. U.S.A..

[36] Schaffter S. W., Scalise D., Murphy T. M., Patel A., Schulman R. (2020). Feedback Regulation
of Crystal Growth by Buffering Monomer Concentration. Nat. Commun..

[37] Ekani-Nkodo A., Kumar A., Fygenson D. K. (2004). Joining
and Scission in the Self-Assembly
of Nanotubes from DNA Tiles. Phys. Rev. Lett..

[38] Lee D. S. W., Choi C.-H., Sanders D. W., Beckers L., Riback J. A., Brangwynne C. P., Wingreen N. S. (2023). Size Distributions of Intracellular
Condensates Reflect Competition between Coalescence and Nucleation. Nat. Phys..

[39] de
Greef T. F. A., Ercolani G., Ligthart G. B. W. L., Meijer E. W., Sijbesma R. P. (2008). Influence of Selectivity on the Supramolecular
Polymerization of AB-Type Polymers Capable of Both A X A and A X B
Interactions. J. Am. Chem. Soc..

[40] Samanta A., Baranda Pellejero L., Masukawa M., Walther A. (2024). DNA-Empowered Synthetic
Cells as Minimalistic Life Forms. Nat. Rev.
Chem.

[41] Weitz M., Kim J., Kapsner K., Winfree E., Franco E., Simmel F. C. (2014). Diversity
in the Dynamical Behaviour of a Compartmentalized Programmable Biochemical
Oscillator. Nat. Chem..

[42] Yamada A., Yamanaka T., Hamada T., Hase M., Yoshikawa K., Baigl D. (2006). Spontaneous Transfer of Phospholipid-Coated Oil-in-Oil and Water-in-Oil
Micro-Droplets through an Oil/water Interface. Langmuir.

[43] Pontani L.-L., van der Gucht J., Salbreux G., Heuvingh J., Joanny J.-F., Sykes C. (2009). Reconstitution
of an Actin Cortex inside a Liposome. Biophys.
J..

[44] Ben
Trad F., Wieczny V., Delacotte J., Morel M., Guille-Collignon M., Arbault S., Lemaître F., Sojic N., Labbé E., Buriez O. (2022). Dynamic Electrochemiluminescence Imaging of Single
Giant Liposome Opening at Polarized Electrodes. Anal. Chem..

[45] Li Y., Maffeo C., Joshi H., Aksimentiev A., Ménard B., Schulman R. (2022). Leakless End-to-End Transport of
Small Molecules through Micron-Length DNA Nanochannels. Sci. Adv..

[46] Thomsen R. P., Malle M. G., Okholm A. H., Krishnan S., Bohr S. S.-R., Sørensen R. S., Ries O., Vogel S., Simmel F. C., Hatzakis N. S., Kjems J. A. (2019). A large size-selective
DNA nanopore with sensing applications. Nat.
Commun..

[47] Liu X., Zhao Y., Liu P., Wang L., Lin J., Fan C. (2019). Biomimetic DNA Nanotubes:
Nanoscale Channel Design and Applications. Angew.
Chem., Int. Ed. Engl..

[48] Dey S., Fan C., Gothelf K. V., Li J., Lin C., Liu L., Liu N., Nijenhuis M. A. D., Saccà B., Simmel F. C., Yan H., Zhan P. (2021). DNA Origami. Nat. Rev. Methods Primers.

[49] Heuer-Jungemann A., Liedl T. (2019). From DNA Tiles to Functional DNA Materials. Trends Chem..

[50] Woods D., Doty D., Myhrvold C., Hui J., Zhou F., Yin P., Winfree E. (2019). Diverse and Robust
Molecular Algorithms Using Reprogrammable
DNA Self-Assembly. Nature.

[51] Zhang D. Y., Hariadi R. F., Choi H. M. T., Winfree E. (2013). Integrating DNA Strand-Displacement
Circuitry with DNA Tile Self-Assembly. Nat.
Commun..

[52] McKinney, W. Data Structures for Statistical Computing in Python. Proceedings of the 9th Python in Science Conference; Austin, TX, June 28−July 3, 2010; pp 56−61. 10.25080/Majora-92bf1922-00a

[53] van
der Walt S., Schönberger J.
L., Nunez-Iglesias J., Boulogne F., Warner J. D., Yager N., Gouillart E., Yu T. (2014). scikit-image: image processing in Python. PeerJ.

