# Supplementary material for: Nanotubes Growth by Self-Assembly of DNA Strands at Room Temperature
Source: ACS Nano. 2025 May 8;19(19):18203–13. doi: 10.1021/acsnano.4c17516 (PMC12120994; doi:10.1021/acsnano.4c17516)
Supplement: Supplementary file 1 [file nn4c17516_si_001.pdf]

Supplementary information for:

Nanotubes growth by self-assembly of DNA strands at room temperature

Laura Bourdon,[1,†] Syed Pavel Afrose,[2,†] Siddharth Agarwal,[2] Debajyoti Das,[3,4] Rajat Singh,[3,4,5] Aurélie Di Cicco,[6] Daniel Lévy,[6] Ayako Yamada,[1] Damien Baigl,[1,*] Elisa Franco[2,*]

[1]PASTEUR, Department of Chemistry, École Normale Supérieure, PSL University, Sorbonne Université, CNRS, 75005 Paris, France

[2]Department of Mechanical and Aerospace Engineering, University of California at Los Angeles, 420 Westwood Plaza, Los Angeles, CA 90095, USA

[3]Department of Medicine, David Geffen School of Medicine, University of California, Los Angeles, Los Angeles, CA 90095, USA

[4]Division of Digestive Diseases, David Geffen School of Medicine, University of California, Los Angeles, Los Angeles, CA 90095, USA

[5]Comprehensive Liver Research Center at University of California, Los Angeles, Los Angeles, CA 90095, USA

[6]Institut Curie, Université PSL, Sorbonne Université, CNRS UMR168, Laboratoire Physico-Chimie Curie, 75005, Paris, France

[†]These authors contributed equally

[*]Corresponding authors: damien.baigl@ens.psl.eu, efranco@seas.ucla.edu

Content:

1. Materials
2. Supplementary figures S1 – S20
3. Legends of the supplementary movies S1 – S5
4. Supplementary references

1. Materials

DNA oligonucleotides

PAGE purified DNA oligonucleotides were purchased from Sigma Aldrich (experiments at ENS) or Integrated DNA Technologies (experiments at UCLA) and resuspended in pure water. The sequences of the five strands required to assemble tiles are taken from [1] and [2], and are presented below preserving the same nomenclature.

Sequences of the single tile design:

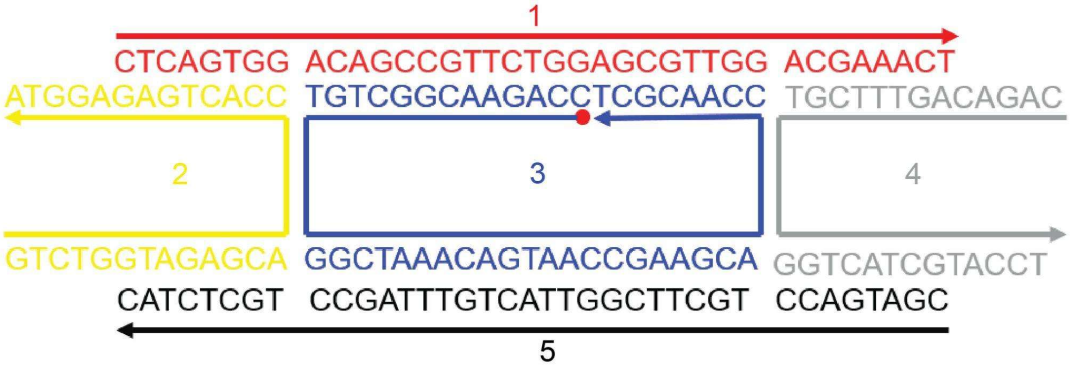

Tile SEs - single tile nanotube assembly

5bSE1	5'- CTC AGT GGA CAG CCG TTC TGG AGC GTT GGA CGA AAC T
5bSE2	5'- GTC TGG TAG AGC ACC ACT GAG AGG TA
5bSE3-Cy3	5'- cy3 - CCA GAA CGG CTG TGG CTA AAC AGT AAC CGA AGC ACC AAC GCT
5bSE4	5'- CAG ACA GTT TCG TGG TCA TCG TAC CT
5bSE5	5'- CGA TGA CCT GCT TCG GTT ACT GTT TAG CCT GCT CTA C

Sequences of the two-tile design:

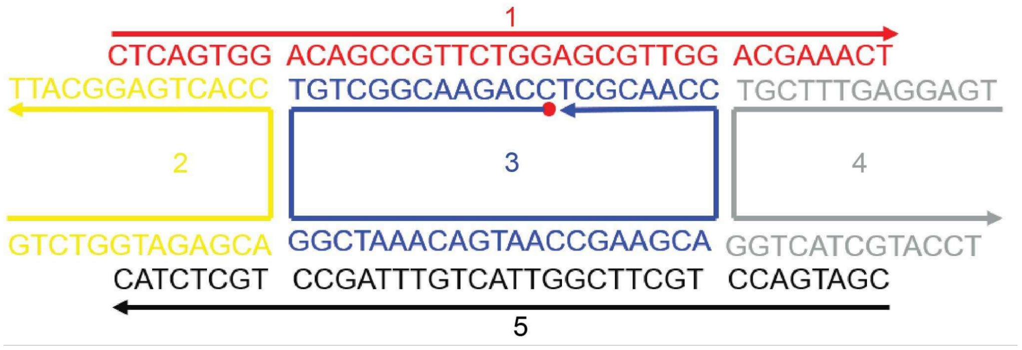

Tile SEp: two-tile nanotube assembly

5bSEp1 5'- CTC AGT GGA CAG CCG TTC TGG AGC GTT GGA CGA AAC T

5bSEp2 5'- GTC TGG TAG AGC ACC ACT GAG GCA TT

5bSEp3-Cy3 5'-cy3-CCA GAA CGG CTG TGG CTA AAC AGT AAC CGA AGC ACC AAC GCT

5bSEp4 5'- TGA GGA GTT TCG TGG TCA TCG TAC CT

5bSEp5 5'- CGA TGA CCT GCT TCG GTT ACT GTT TAG CCT GCT CTA C

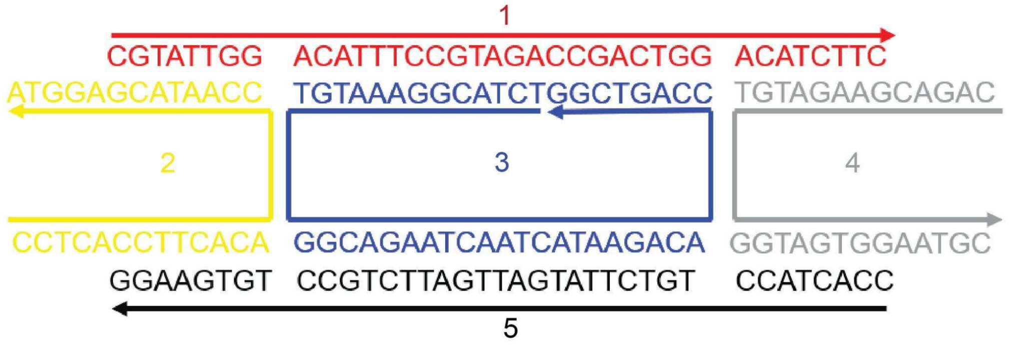

Tile REp: two-tile nanotube assembly

5bREp1 5'- CGT ATT GGA CAT TTC CGT AGA CCG ACT GGA CAT CTT C

5bREp2 5'- CCT CAC CTT CAC ACC AAT ACG AGG TA

5bREp3 5'- TCT ACG GAA ATG TGG CAG AAT CAA TCA TAA GAC ACC AGT CGG

5bREp4 5'- CAG ACG AAG ATG TGG TAG TGG AAT GC

5bREp5 5'- CCA CTA CCT GTC TTA TGA TTG ATT CTG CCT GTG AAG G

Lipids

The lipids 1,2-dioleoyl-sn-glycero-3-phosphocholine (18:1 (Δ9-Cis) PC (DOPC)) and 1,2-dioleoyl-sn-glycero-3-phosphoethanolamine-N-(lissamine rhodamine B sulfonyl) (18:1 Liss Rhod PE) were delivered as a powder form (Avanti Polar lipids, USA). Stock solutions of 20 $mg.mL^{-1}$ DOPC, and 1 $mg.mL^{-1}$ Liss Rhod PE were prepared in $CHCl_3$ and stored at -25°C until use.

Oil and Surfactant

An immiscible fluorocarbon oil Fluorinert™ FC-40, was used to make the water-in-oil droplets. Fluorinert™ FC-40 was purchased from Sigma-Aldrich™ (CAS Number 86508-42-1 MDL number MFCD00131095). For droplet encapsulation, RAN Biotech's non-ionic surfactant (cat#: 008-FluoroSurfactant-) was used, which is a linear poly(ethylene glycol), MW ca. 600, coupled on each end to Krytox-FSH via an amide group.

The oil-surfactant mix consists of FC-40 fluorinated oil and 2% w/v perfluoropolyether-polyethylene glycol (PFPE-PEG) block-copolymer fluorosurfactant with Krytox-FSH via an amide group (Ran Biotechnologies).

2. Supplementary figures

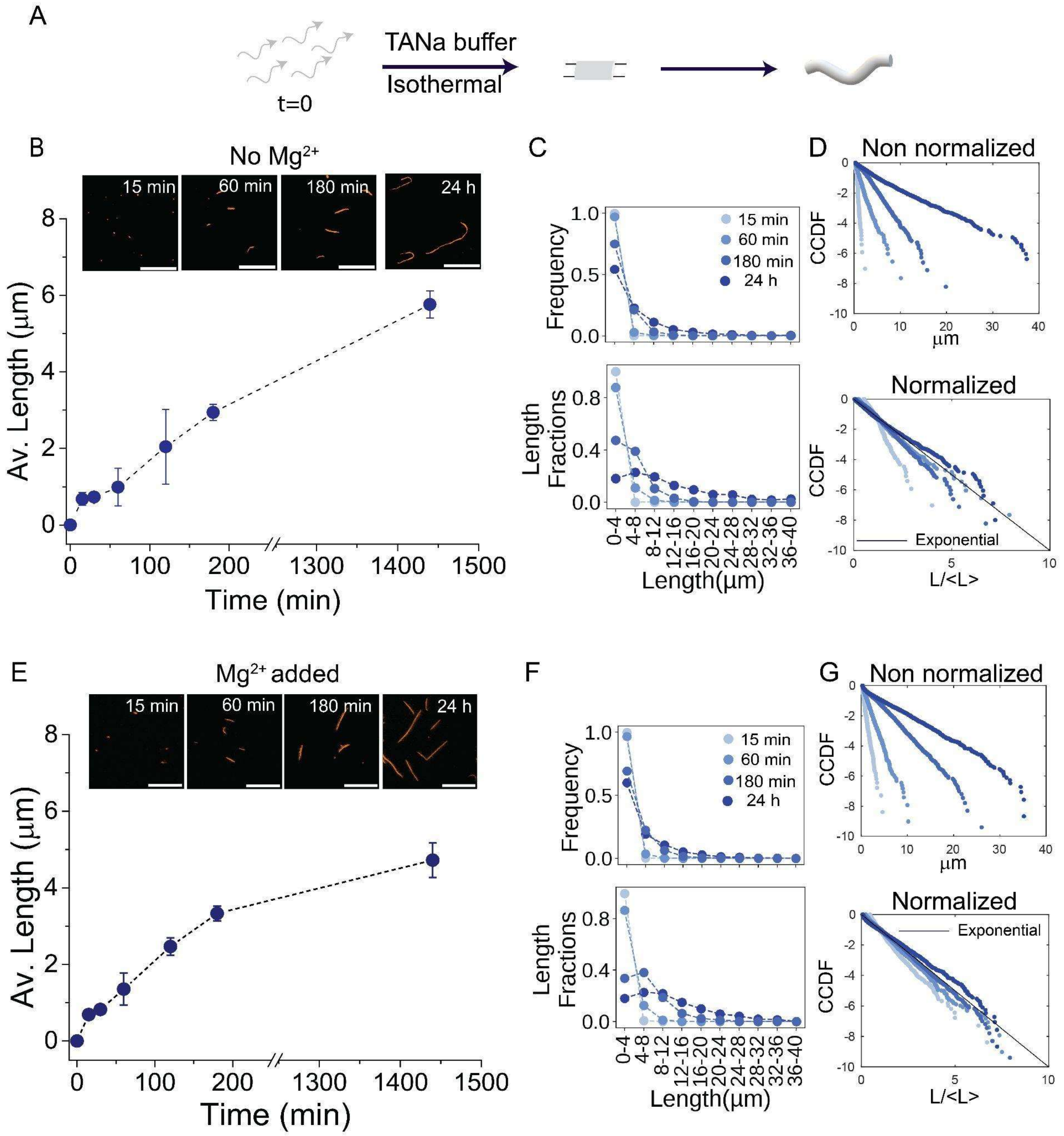

Figure S1. A) Scheme showing the isothermal assembly process in TANa buffer when all 5 strands were added together, B) microscopy images and average nanotube length at different time points, and C) frequency and length fraction histograms. Total nanotubes analyzed across triplicates at each time point: 0 min: 0, 15 min: 2210, 30 min: 3195, 60 min: 1049, 120 min: 838, 180 min: 1863, 24 h: 1480. D) Cumulative complementary distribution function plots. E) Microscopy images and average nanotube length of the same system at different time points when 5 mM Mg^{2+} was added at each time point before imaging, F) frequency and length fraction histograms. Total nanotubes analyzed across triplicates at each time point: 0 min: 0, 15 min: 2196, 30 min: 5470, 60 min: 4143, 120 min: 4045, 180 min: 6019, 24 h: 2903. G) Cumulative complementary distribution function plots. Error bars represent standard deviations of the mean of triplicate experiments. Scale bars = 10 μm.

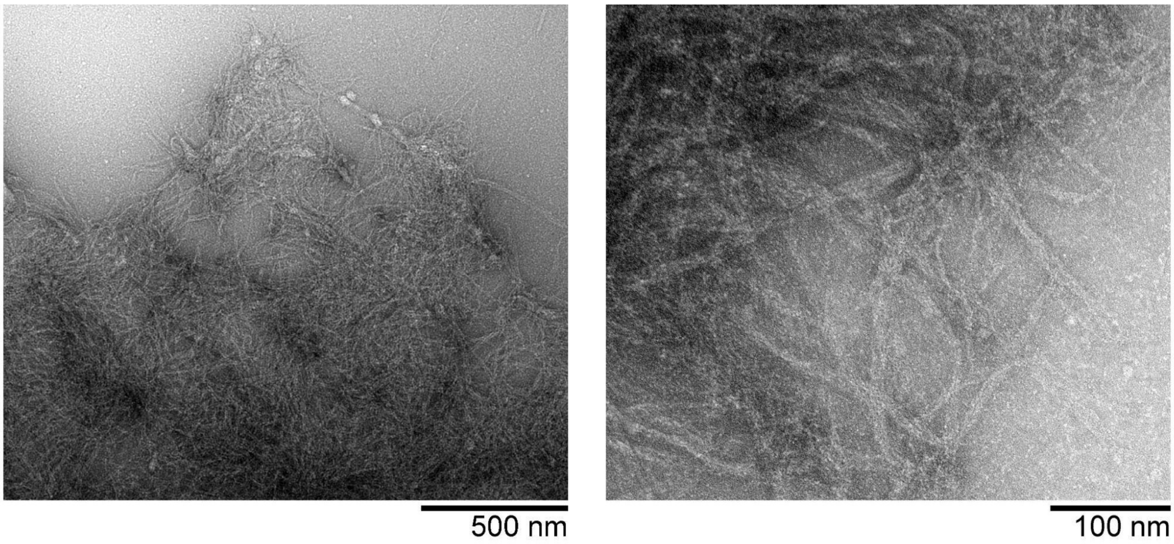

Figure S2. Transmission electron microscopy images of DNA nanotubes obtained by isothermal assembly at room temperature in TAEMg.

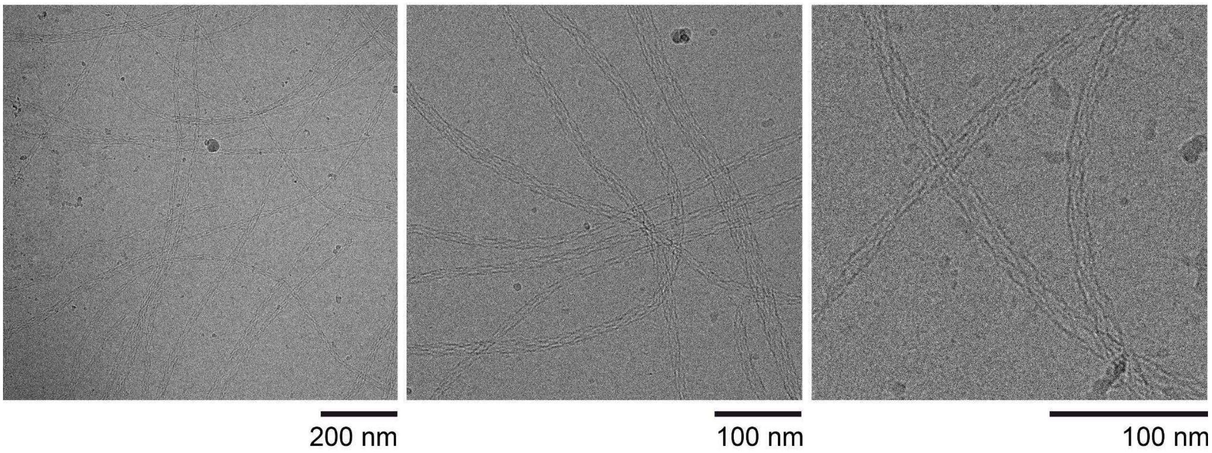

Figure S3. Cryo-electron microscopy images of DNA nanotubes obtained by thermal annealing in TAMg ([$MgCl_2$] = 12.5 mM).

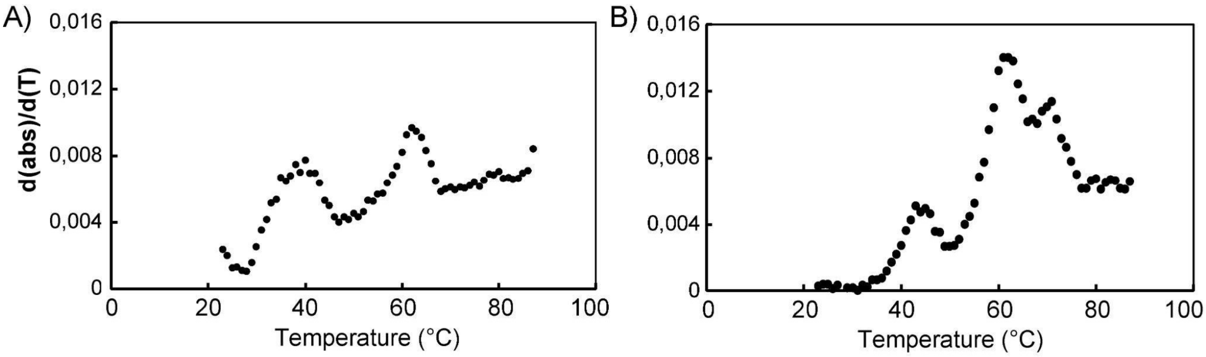

Figure S4. Melting curves of DNA nanotubes formed A) isothermally in TANa for 4 days at room temperature, and B) by thermal annealing in TAEMg. Each plot displays the derivative of absorbance at 260 nm with temperature, as a function of temperature.

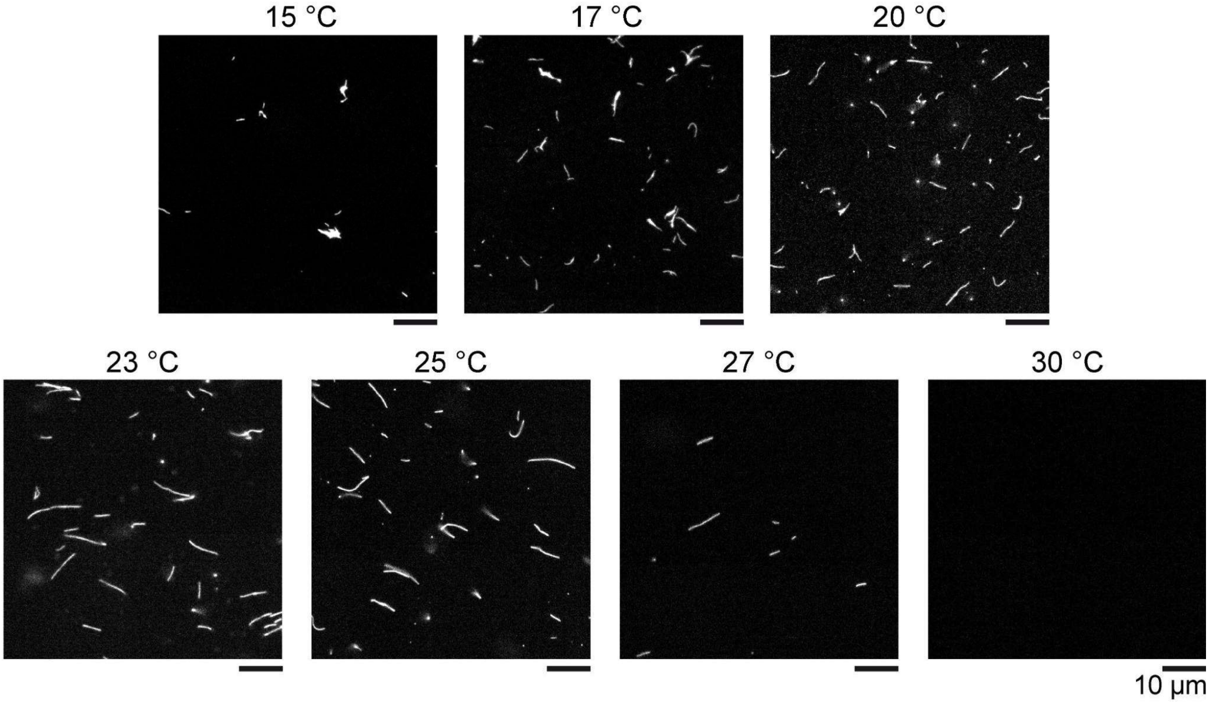

Figure S5. Fluorescence microscopy images after 1 day of DNA self-assembly in TANa at a fixed temperature ranging from 15 °C until 30 °C.

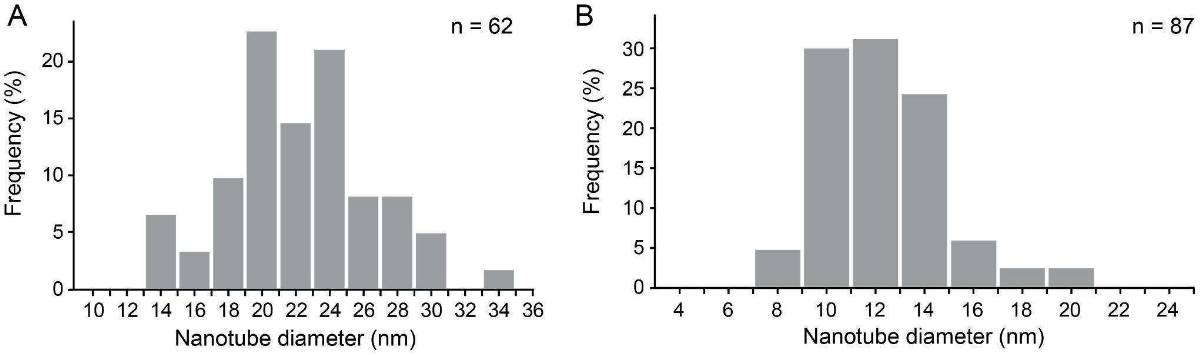

Figure S6. Histograms of DNA nanotube diameters measured from Cryo-EM images. A) Nanotubes self-assembled in TANa ([NaCl] = 100 mM) at room temperature for 10 days. B) Nanotubes self-assembled in TAMg ([$MgCl_2$] = 12.5 mM) by thermal annealing.

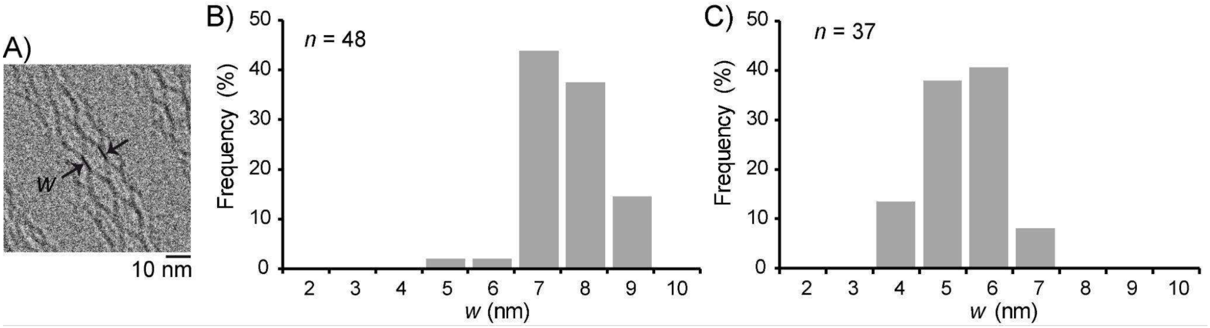

Figure S7. Tile width distribution from cryo-EM images of DNA nanotubes. A) Definition of the measured width *w*. B-C) Width distribution of nanotubes B) self-assembled in TANa for 5 h at room temperature, and C) self-assembled by thermal annealing in TAMg buffer.

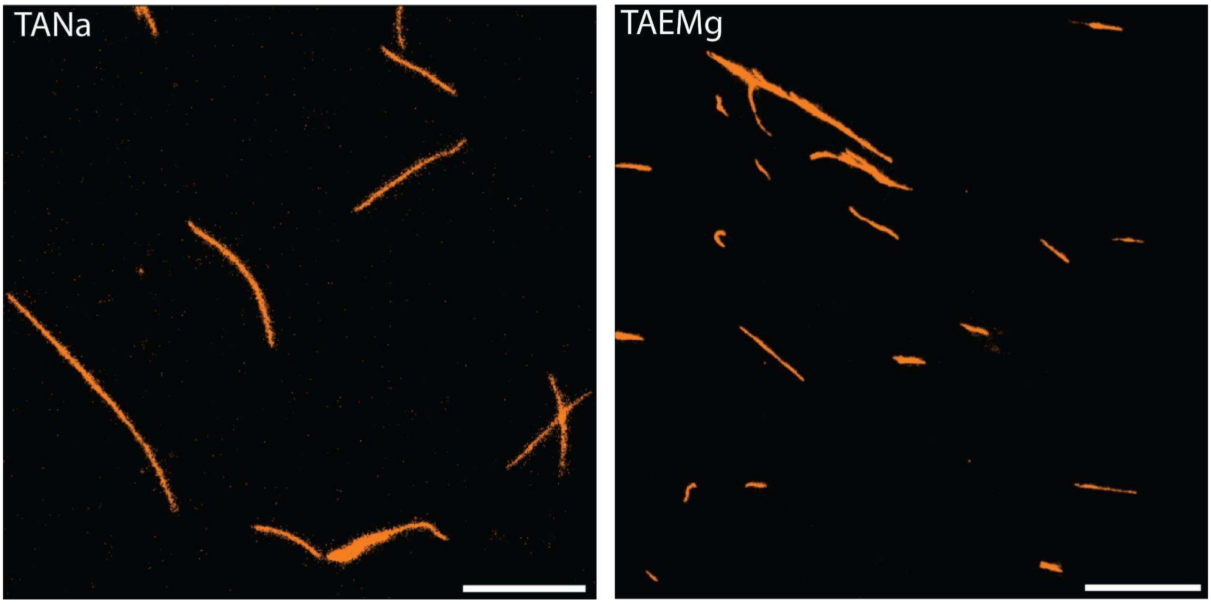

Figure S8. Nanotubes obtained from the single tile design self-assembled for 1 day at room temperature in TANa (left image) or in TAEMg buffers (right image). In TANa and in TAEMg, assembly was carried out with pre-assembled tile with four strands where the fifth strand (SEp4) was added isothermally. Scale bar = 10 µm.

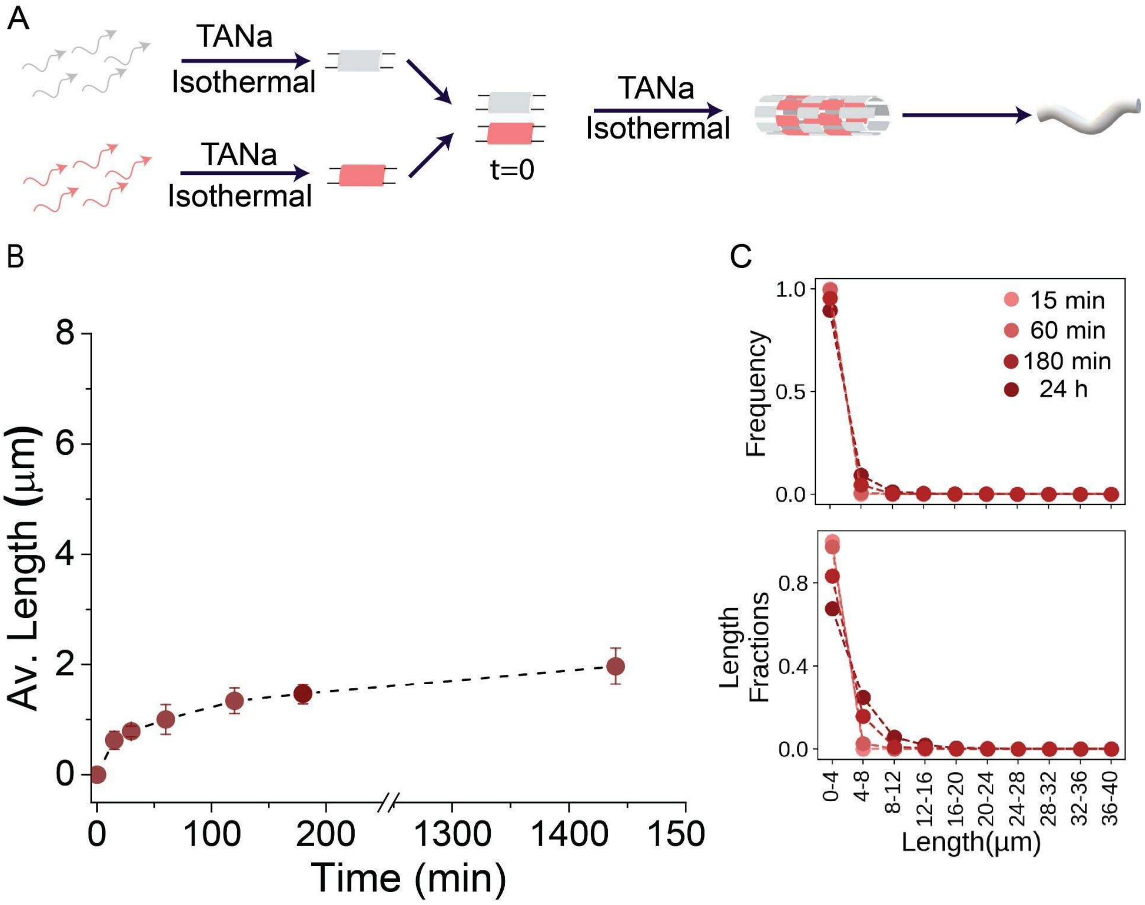

Figure S9. A) Scheme showing the isothermal formation of two-tile nanotubes in TANa, B) average nanotube length at different time points, and C) frequency and length fraction histograms. Total nanotubes analyzed across triplicates at each time point: 0 min: 0, 15 min: 145, 30 min: 106, 60 min: 876, 120 min: 1945, 180 min: 2888, 24 h: 14853. Error bars represent standard deviations of the mean of triplicate experiments.

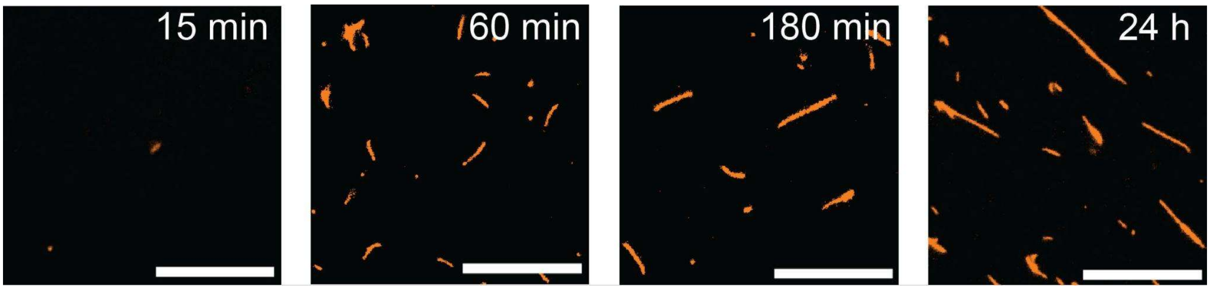

Figure S10. Microscopy images of two-tile nanotubes in TANa at different time points. Scale bars = 10 µm.

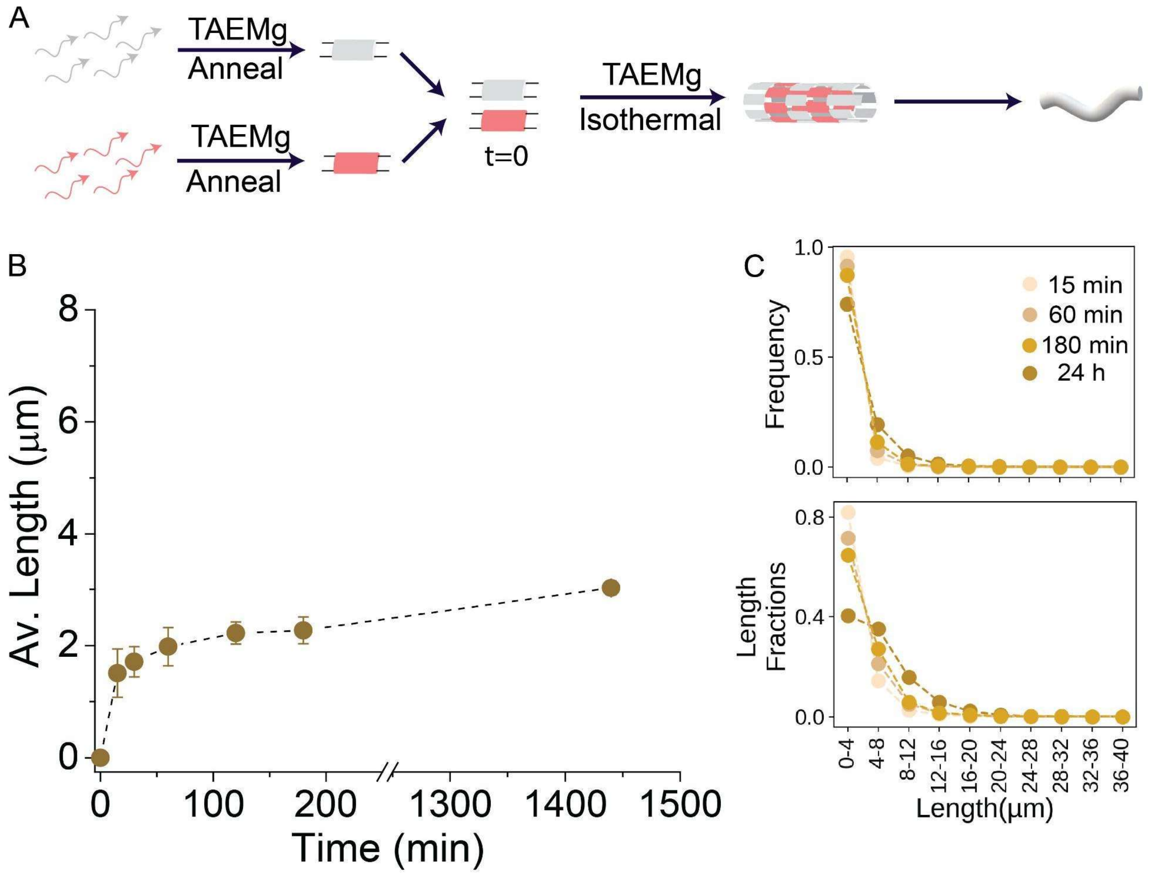

Figure S11. A) Scheme showing the formation of two-tile nanotubes in TAEMg, B) average nanotube length at different time points, and C) frequency and length fraction histograms. Total nanotubes analyzed across triplicates at each time point: 0 min: 0, 15 min: 29380, 30 min: 25390, 60 min: 25761, 120 min: 22240, 180 min: 16714, 24 h: 6648. Error bars represent standard deviations of the mean of triplicate experiments.

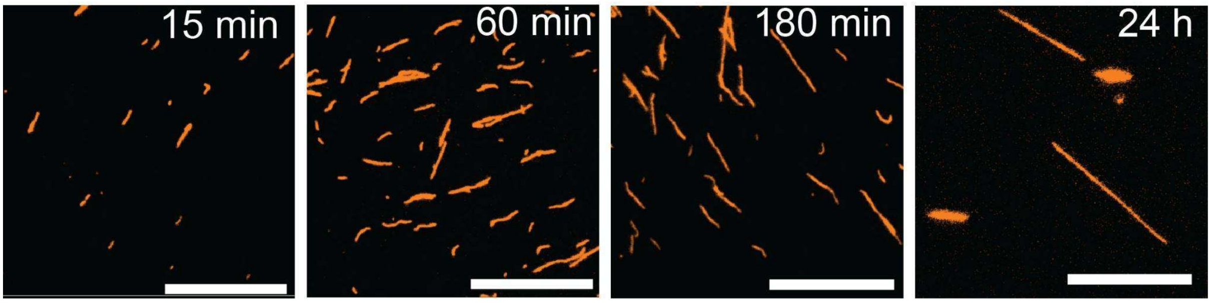

Figure S12. Microscopy images of two-tile nanotubes in TAEMg at different time points. Scale bars = 10 μm.

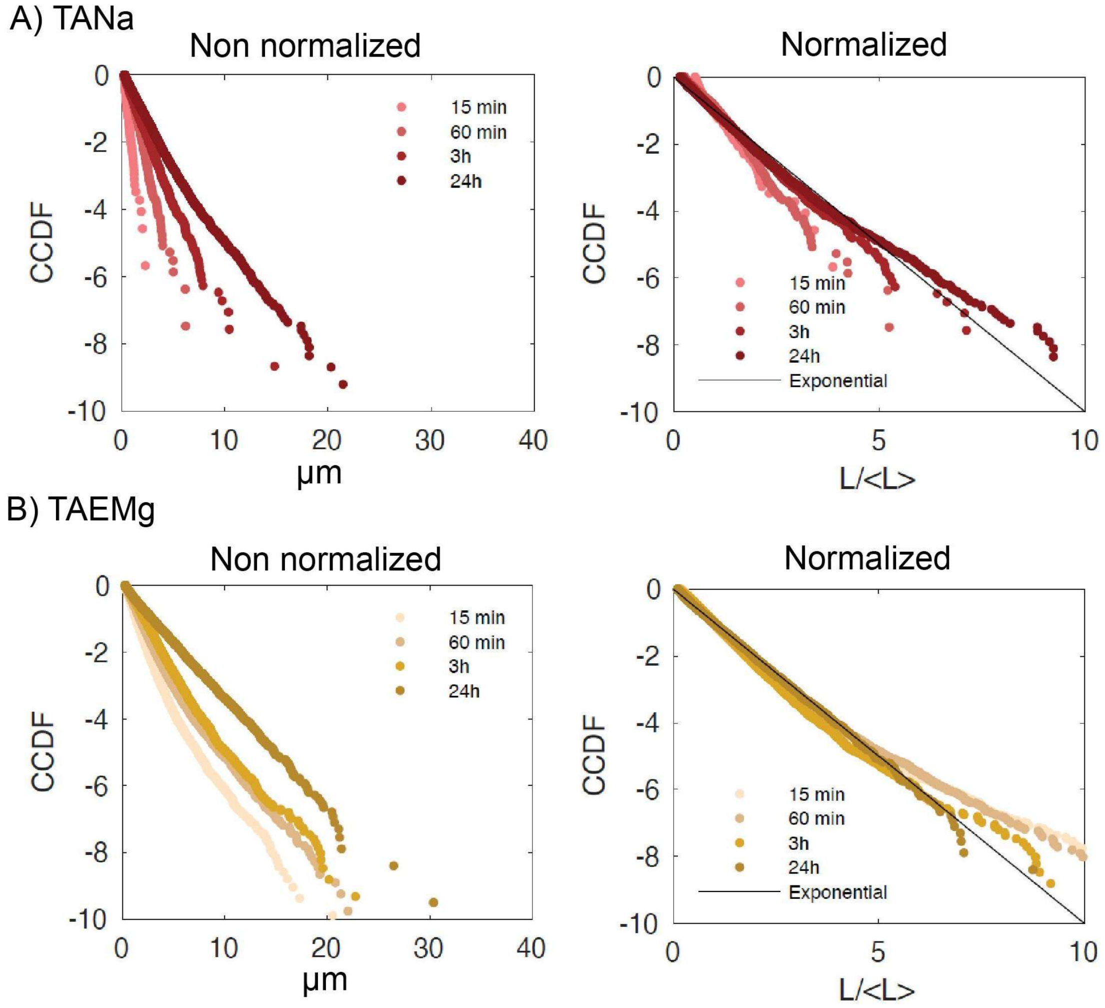

Figure S13. Cumulative complementary distribution function plots for two-tile nanotubes in TANa and TAEMg buffer.

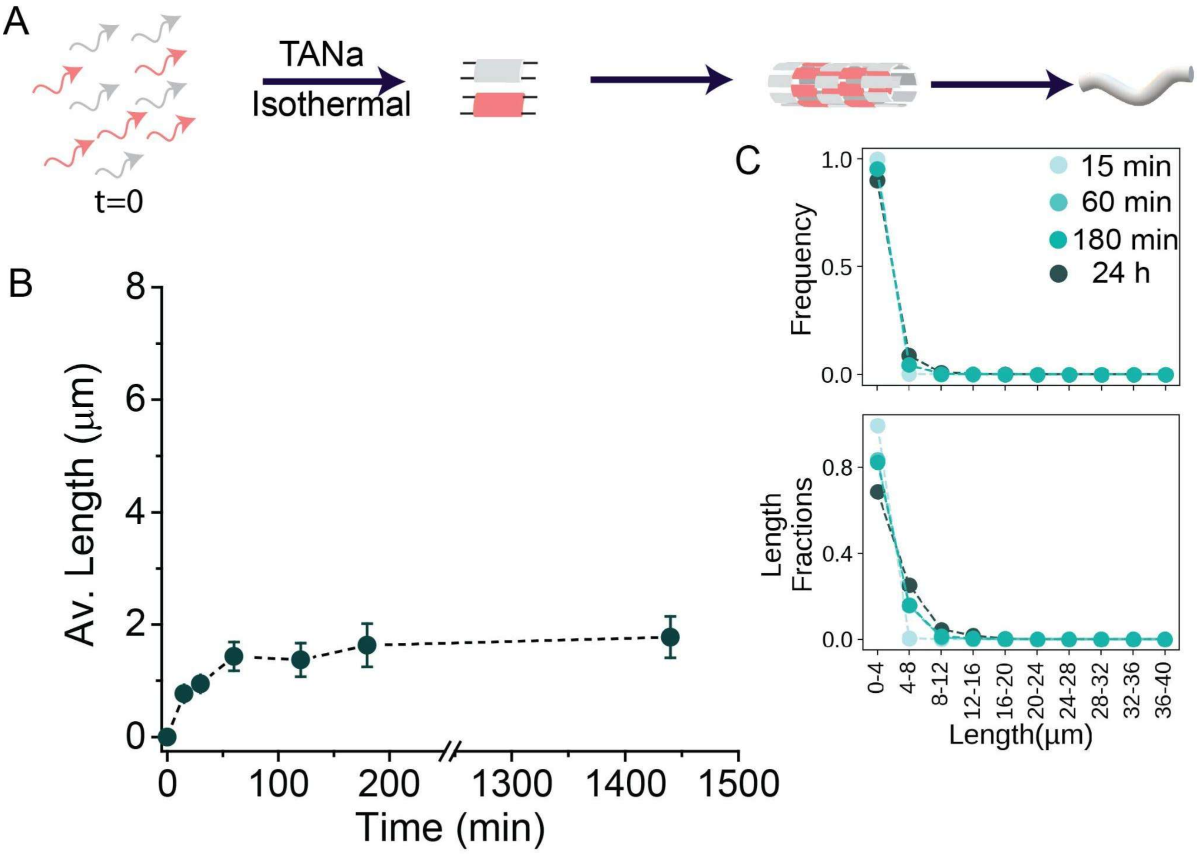

Figure S14. A) Scheme showing the formation of two-tile design nanotubes in TANa when all the ten strands were added together without prior incubation. B) average nanotube length at different time points, and C) frequency and length fraction histograms. Total nanotubes analyzed across triplicates at each time point: 0 min: 0, 15 min: 14375, 30 min: 5400, 60 min: 6536, 120 min: 5937, 180 min: 9401, 24 h: 8888. [strands] = 1 µM. Error bars represent standard deviations of the mean of triplicate experiments.

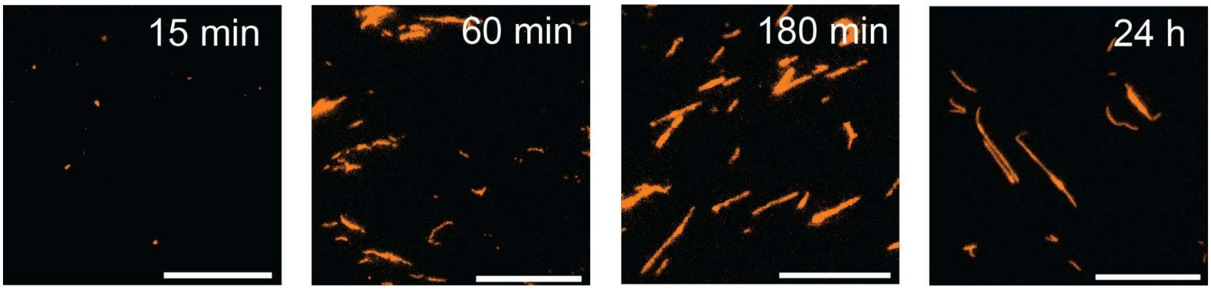

Figure S15. Representative fluorescence microscopy images of nanotubes made from two-tile designs in TANa over time when all the ten strands were added together without prior incubation. [strands] = 1 µM. Scale bars = 10 µm.

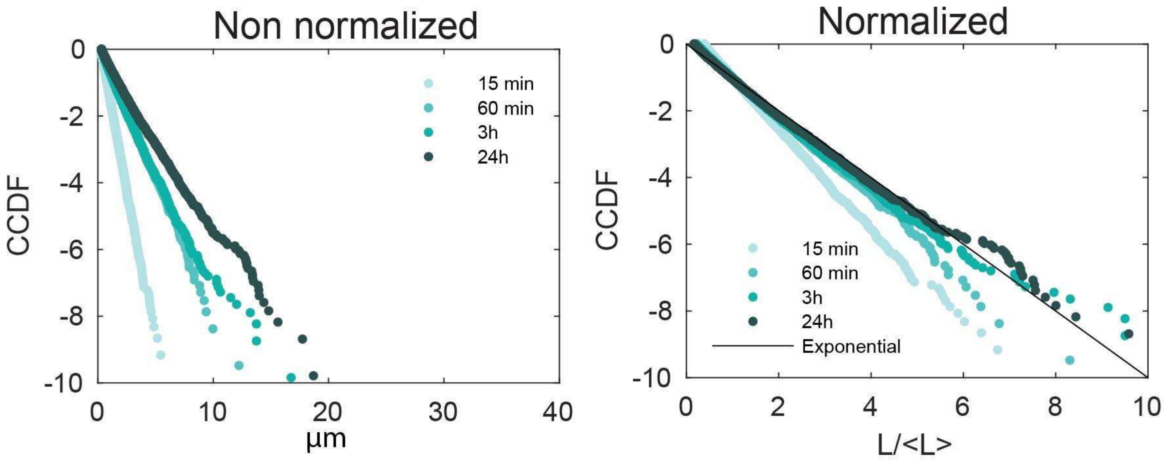

Figure S16. Cumulative complementary distribution function plots for two-tile nanotubes in TANa buffer when all ten strands were added without incubation.

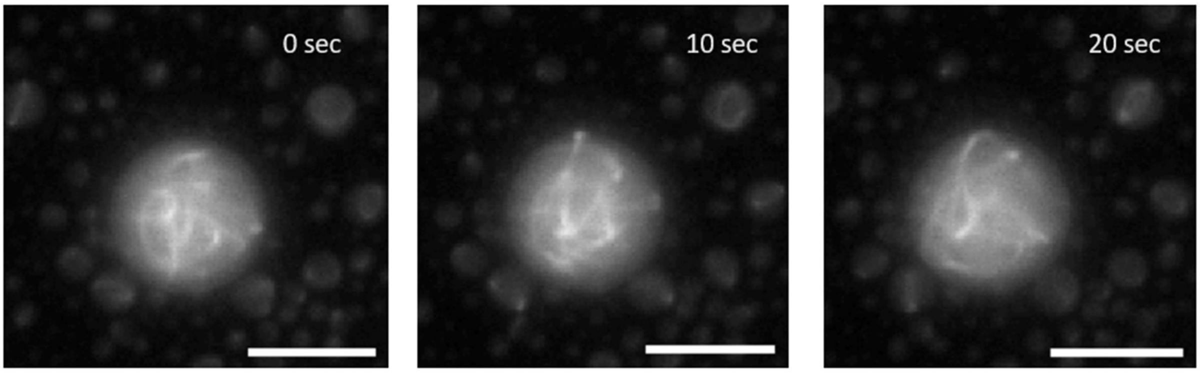

Figure S17. Fluorescence microscopy images of single tile nanotubes made in TANa buffer inside water-in-oil droplets at different times showing the dynamic nature of the nanotubes. Scale bars = 20 µm.

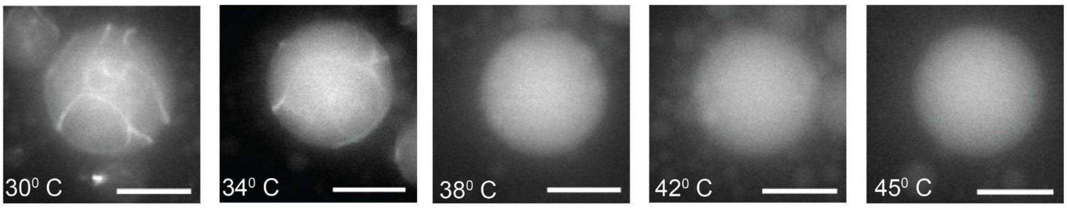

Figure S18. Representative microscopy images of single nanotubes formed after two-days of isothermal assembly in water-in-oil droplets and exposed to different temperatures. Scale bars = 20 µm. Each strand concentration was 100 nM.

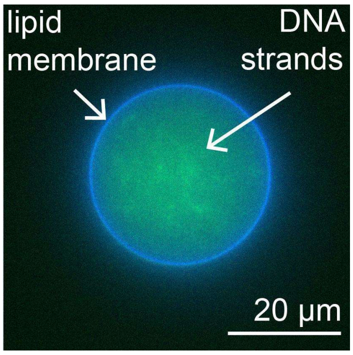

Figure S19. Representative fluorescence microscopy image of a DOPC/Liss Rhod PE vesicle (in blue) encapsulating DNA strands from the one-tile nanotube design (in green). The image was taken 1 day after the vesicle formation.

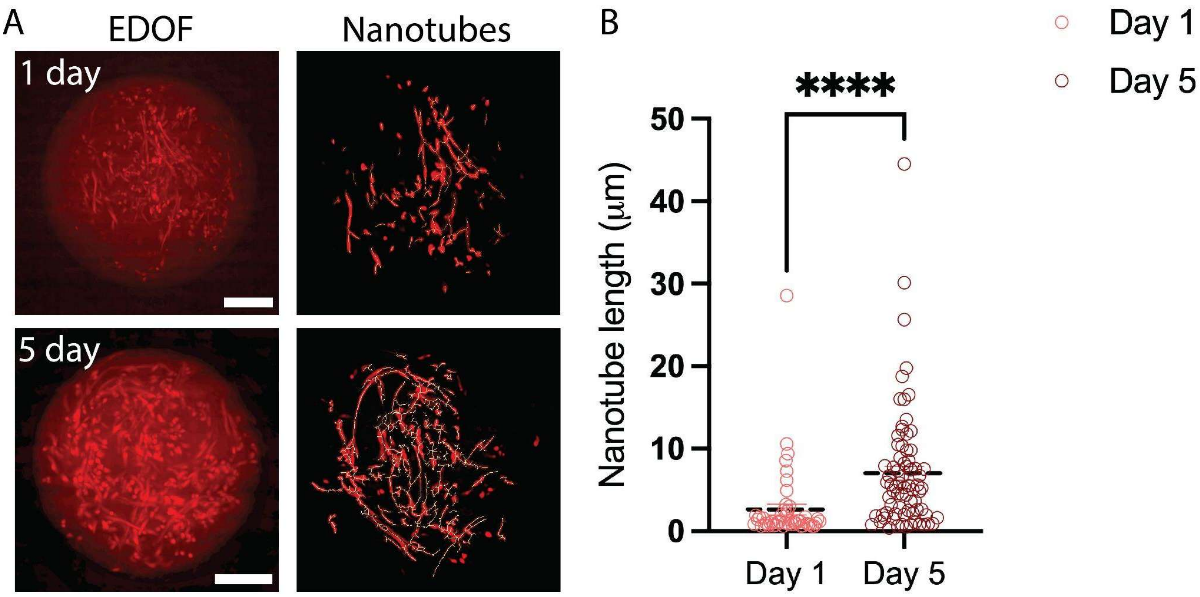

Figure S20. A) Spinning disk confocal images of DNA nanotubes inside the same GUV at day 1 (top) and day 5 (bottom). The extended depth of focus (EDOF) images along with the 3D reconstruction of the nanotubes inside the vesicles show an intensification of the nanotube network. B) Comparative distribution of the apparent nanotube lengths inside the vesicles at day 1 and day 5. Non parametric unpaired t-test, *P-value<0.05. As explained in the Methods section, this graph does not represent the accurate distribution of the lengths of each nanotube but faithfully assesses the global evolution of the nanotube length distribution over time (see Methods for details on the image analysis). Scale bar = 10 µm. Z Steps: 0.20 µm, No. of Steps: 425 (1 day) and 363 (5 day). Vesicle diameter: 42.96 µm (1 day), 42.86 µm (5 day).

3. Legends of the supplementary movies

Movie S1. Real-time fluorescence video of the self-assembly of DNA nanotubes in TANa buffer at room temperature.

Movie S2. Real-time fluorescence video of DNA assemblies obtained from the one-tile design in TAMg buffer at room temperature for 10 days.

Movie S3. Real-time fluorescence video of the self-assembly and self-organization of DNA nanotubes inside water-in-oil droplets at room temperature.

Movie S4. Real-time fluorescence video of the self-assembly and self-organization of DNA nanotubes inside giant unilamellar vesicles at room temperature.

Movie S5. 3D reconstruction of nanotubes encapsulated inside a GUV on day 1 and day 5 across the z- axis.

4. Supplementary References

[1] P. W. K. Rothemund, A. Ekani-Nkodo, N. Papadakis, A. Kumar, D. K. Fygenson, E. Winfree, *J. Am. Chem. Soc.* **2004**, *126*, 16344–16352.
[2] S. Agarwal, M. A. Klocke, P. E. Pungchai, E. Franco, *Nat. Commun.* **2021**, *12*, 3557.
